# Unexpected Evolutionary Divergence of Tachykinin‐Positive Neurons Innervating the Central Complex in Hexapods

**DOI:** 10.1002/cne.70142

**Published:** 2026-02-24

**Authors:** Uwe Homberg, Jing Xu, Tim Keyser, Fedor Suzdalenkov, Fabian Wimmer, Martina Fromandi, Stefano Bianco, Michelle Tez, Stefan Dippel

**Affiliations:** ^1^ Department of Biology, Animal Physiology Philipps‐Universität Marburg Marburg Germany; ^2^ Center for Mind, Brain and Behavior (CMBB) Marburg University and Justus Liebig University Giessen Marburg Germany

**Keywords:** central body, hexapod phylogeny, immunocytochemistry, insect brain, neuropeptide, protocerebral bridge

## Abstract

The central complex comprises an assemblage of midline‐spanning neuropils in the brain of insects that play a key role in goal‐directed orientation and navigation vector calculation. The central complex consists of layers of tangential input neurons that contact topographically organized columnar neurons which provide outputs to the right and left brain hemispheres. Its anatomical organization and functional role are regarded as highly conserved across insects. In addition to classical neurotransmitters, a wide range of neuropeptides have been detected in the central complex including peptides of the tachykinin family. Because the cellular identity of tachykinin‐containing neurons in the central complex has not been determined in most cases, we used antisera against tachykinin I and II from the migratory locust, termed Lom‐TKs, to identify the immunolabeled neurons in hexapods ranging from flightless two‐pronged bristletails to flies. The data show that LomTK‐related peptides are present in the central complex of all studied species except crickets. In most species one or several types of columnar neurons were immunolabeled, sometimes together with certain subsystems of tangential input neurons. The types of immunolabeled columnar neurons, however, are distinctly different between species from different orders, in some cases even between insects within the same order, and comprise cell types that innervate either the upper or lower division of the central body. This high degree of evolutionary divergence of tachykinin‐positive neurons, even in closely related groups, may be related to species‐specific differences in navigational requirements and calls for caution with respect to homologizing neurons across clades.

## Graphical abstract

The central complex is a group of midline‐spanning neuropils in the brain of insects with a key role in goal‐directed orientation and navigation. Immunolabeling in 25 species, ranging from bristletails to flies, shows that neurons containing peptides of the tachykinin family of neuropeptides are present in most species studied. Often, sets of columnar neurons show immunostaining, but the types of labeled neurons differ considerably, possibly related to species‐specific differences in navigational requirements.

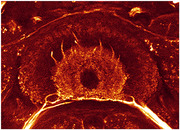

## 1 Introduction

Tachykinins are one of the largest families of signaling peptides with members occurring in all major bilaterian clades (Maggio 1988; Nässel 1999; Severini et al. 2002; Steinhoff et al. 2014; Nässel et al. 2019). Most tachykinins in deuterostomes are characterized by a conserved—FXGLMamide carboxy terminus, exemplified by substance P, the archetypical tachykinin member, whereas tachykinin‐related peptides in protostomes usually have the more flexible—FX_1_GX_2_Ramide carboxyl end, with X_1_ and X_2_ being variable amino acids (Table 1). Tachykinins serve a plethora of different functions as signaling molecules outside and inside the nervous system. In vertebrates, substance P and other tachykinins are involved in pain perception, stress, aggression, inflammation, hormone release, and various brain circuits and, outside the nervous system, serve roles in gut motility, ion transport, and fluid secretion (Nässel et al. 2019). Amphibian skin secretions are particularly rich in tachykinins and likely playing a defensive role.

**TABLE 1:** Sequence alignment of selected tachykinin‐related peptides from the studied hexapods in comparison to LomTK‐I and LomTK‐II.

Hexapod species	Designation	Sequence	Reference
*Locusta migratoria*	LomTK‐I	**GP SGFYGVRa**	Schoofs et al. (1990)
	LomTK‐II	**APL SGFYGVRa**	Schoofs et al. (1990)
*Campodea augens*		[**TPDRFFGMRa**]**^1^**	Derst et al. (2016)
*Thermobia domestica*		**VSNGFYGVRa**	Derst et al. (2016)
*Platycnemis pennipes*		**AP SGFLGMRa**	NCBI, SRR1695275, Kohli et al. (2021)
*Coenagrion puella*		**APSSGFFGMRa**	NCBI, SRR1695222, Kohli et al. (2021)
*Libellula quadrimaculata*		**APNSGFFGMRa**	NCBI, SRR1695256 and SRR12518610, Kohli et al. (2021)
*Schistocerca gregaria*	TKRP‐1	**APLLGFHGVRa**	Clynen and Schoofs (2009)
*Acheta domesticus*		**not present**	
*Medauroidea extradentata*		**APSSGFLGLRa**	NCBI, GAWD01015108, Shelomi et al. (2014)
*Rhyparobia maderae*	LemTRP‐1	**AP SGFLGVRa**	Muren and Nässel (1996)
*Hierodula membranacea*		[**AP SGFLGVRa**]**^2^**	NCBI, SRR13888807
*Notonecta glauca*		**GP SGFMGVRa**	NCBI, ERR10774013, Fischer et al. (2023)
*Graphosoma italicum*		**APAAGFFGMRa**	NCBI, ERR14835886, Biodiversity Genomics Europe project
*Apis mellifera*		**ALMGFQGVRa**	Takeuchi et al. (2003)
*Vespula germanica*		**APMGFQGMRa**	OrthoDB, www.orthodb.org/?gene=30212_0:001407
*Vespula vulgaris*		**APMGFQGMRa**	NCBI, XP_050853719.1
*Tribolium castaneum*	Trica‐TRP‐1	**AP SGFTGVRa**	Yeoh et al. (2017)
*Zophobas morio*		**AP SGFTGVRa**	NCBI, KAJ3646212.1, Kaur et al. (2023)
*Acilius sulcatus*		**n/a**	
*Gyrinus substriatus*		**n/a**	
*Ilybius fuliginosus*		**n/a**	
*Manduca sexta*	Manse‐TRP‐1	**LPQGFVGMRa**	Kanost et al. (2016)
*Agrotis ipsilon*	TK‐6	**KAQMGFFGMRa**	Diesner et al. (2018)
*Calliphora vicina*		**APTAFYGVRa**	NCBI, XP_065370124.1
*Drosophila melanogaster*	Drome‐TRP‐2	**APLAFVGLRa**	Flybase.org
*Tipula paludosa*		[**AP SGFVGMRa**]**^3^**	NCBI, GCA_963932295

*Note:* For each species, only the neuropeptide most similar to LomTK‐I and LomTK‐II is presented. Amino acid residues identical to both LomTK‐I and LomTK‐II are highlighted in red, those identical to either LomTK‐I or LomTK‐II are highlighted in blue. Sequences in brackets are from related species: ^1^ *Lepidocampa weberi*, ^2^ *Hierodula patellifera*, ^3^ *Tipula lateralis*.

In insects, tachykinins are widely distributed in the nervous system and gut (Nässel 1999; Nässel et al. 2019). In various species, tachykinins have myostimulatory effects on the heart, oviduct, fore‐ and hindgut (Schoofs et al. 1993; Nässel 1999; Sliwowska et al. 2001). In locusts, tachykinins stimulate fluid secretion of Malpighian tubules (Johard et al. 2003) and, when released from lateral neurosecretory cells of the brain, induce the release of adipokinetic hormone from the corpora cardiaca (Nässel et al. 1995). Various functions were established for tachykinins within the nervous system. In the antennae and/or the antennal lobes of several insect species, tachykinins modulate odor perception (Winther et al. 2006; Ignell et al. 2009; Jung et al. 2013; Ko et al. 2015; Gui et al. 2017). In the fly *Drosophila melanogaster*, male‐specific neurons with wide ramifications in lateral brain areas promote aggression toward conspecific males through the release of tachykinin (Asahina et al. 2014; Wohl et al. 2023). Certain neurons of the central complex (CX) of *Drosophila* regulate fructose‐feeding preference through the release of tachykinins (Musso et al. 2021). Finally, knockdown of tachykinin expression in CX neurons of *Drosophila* resulted in increased center zone avoidance and increased numbers of rest‐activity bouts (Kahsai et al. 2010).

As illustrated by numerous studies in various insect species, the CX plays a pivotal role in spatial orientation, such as goal‐directed navigation, sun‐compass orientation, and spatial memory (reviewed by Pfeiffer and Homberg 2014; Honkanen et al. 2019; Varga et al. 2017; Homberg, Hensgen, et al. 2023; Pfeiffer 2023; Heinze 2023). The CX comprises a group of highly interconnected neuropils spanning the brain midline. Subcomponents are the protocerebral bridge (PB), the upper (CBU) and lower (CBL) divisions of the central body, also termed fan‐shaped body (FB) and ellipsoid body (EB), respectively, and a pair of globular noduli (Pfeiffer and Homberg 2014). The PB, CBU, and CBL consist of arrays of 16–18 columns that are interconnected in a highly topographic manner by columnar and pontine neurons. In addition, the CBU, CBL, and NO show a layered organization, largely determined by the arborization domains of tangential input neurons (Pfeiffer and Homberg 2014).

The CX is a hotspot of neuropeptide expression (Loesel et al. 2002; Nässel and Homberg 2006; Kahsai and Winther 2011; Kaiser et al. 2022; Wolff et al. 2025), and the presence of tachykinins in this brain area has been reported in numerous species (blowfly: Lundquist et al. 1994; Madeira cockroach: Muren et al. 1995; mealworm beetle: Wegerhoff et al. 1996; honeybee: Kaiser et al. 2022; desert locust: Vitzthum and Homberg 1998; tobacco budworm: Zhao et al. 2017). As suggested by immunostaining, tachykinins were reported in columnar neurons connecting the PB, CBU, and lateral complex (mealworm beetle: Wegerhoff et al. 1996; cockroach: Muren et al. 1995), columnar neurons connecting the CBU and lateral complex (locust: Vitzthum and Homberg 1998), columnar neurons connecting the PB, CBL, and lateral complex (locust: Vitzthum and Homberg 1998), tangential neurons of the PB and of the CBL (locust: Vitzthum and Homberg 1998), and tangential and columnar neurons of the FB (flies: Lundquist et al. 1994; Nässel 2002; Wolff et al. 2025). Recently, refined anatomical studies, particularly in the fruit fly (Hulse et al. 2021), desert locust (Heinze and Homberg 2008; von Hadeln et al. 2020), and bumblebee (Sayre et al. 2021), have greatly advanced our understanding of neuronal cell types in the insect CX allowing for more specific identification of immunolabeled neurons of the CX than was previously possible. To analyze the distribution and potential evolution of tachykinin‐expressing neurons in the CX in greater detail, we examined tachykinin‐immunolabeled neurons in the CX of various hexapod species, ranging from two‐pronged bristletails (Diplura) to flies (Diptera), with the aim of identifying the labeled cell types as a foundation for future functional studies.

## 2 Materials and Methods

### 2.1 Hexapod Species

Twenty‐five species from 12 hexapod orders were used for the experiments. *Campodea augens* (Diplura), collected from the forest floor near Weilburg, Hesse, was provided by Chris Dlouhy. Firebrats (*Thermobia domestica*), blowflies (*Calliphora vicina*), and praying mantises (*Hierodula membranacea*) were obtained from commercial vendors. Tobacco hawk moths (*Manduca sexta*) were obtained from Dr. Monika Stengl (University of Kassel), black cutworm moths (*Agrotis ipsilon*) from Dr. Christophe Gadenne (INRA, Angers), fruit flies (*D. melanogaster*) from Dr. Jörg Großhans (Marburg University), and honeybees (*Apis mellifera*) from Bieneninstitut Kirchhain. Backswimmers (*Notonecta glauca*), dragonflies (*Libellula quadrimaculata, Platycnemis pennipes*, and *Coenagrion puella*), and diving beetles (*Acilius sulcatus, Gyrinus substriatus*, and *Ilybius fuliginosus*) were obtained from ponds, and shield bugs (*Graphosoma italicum*), crane flies (*Tipula paludosa*), German wasps (*Vespula germanica*), and common wasps (*Vespula vulgaris*) from meadows near the Department of Biology. House crickets (*Acheta domesticus*), walking sticks (*Medauroidea extradentata*), darkling beetles (*Zophobas morio*), red flour beetles (*Tribolium castaneum*), Madeira cockroaches (*Rhyparobia maderae*), and desert locusts (*Schistocerca gregaria*) were obtained from cultures at Marburg University (Department of Biology, Animal Physiology). House crickets were kept at a temperature of 22°C and a humidity of 50%. The cockroaches were kept in a plastic box at a constant temperature of 24°C and a humidity of 50%. The stick insects were kept at room temperature in a terrarium with blackberry branches. The light‐dark rhythm was 12:12 h for all six species.

### 2.2 Immunocytochemistry

Immunostaining was either done on brain sections using the peroxidase‐antiperoxidase (PAP) technique (Sternberger 1979) or on wholemount brains using the indirect immunofluorescence labeling technique. Following cold anesthesia animals were decapitated. Brains were dissected and fixed overnight in 4% paraformaldehyde in phosphate buffered saline (PBS, 0.01 mol L^−1^, pH 7.4).

For immunostaining following the PAP technique, brains were embedded in gelatin–albumin and sectioned into 30‐µm slices using a vibrating blade microtome (Leica VT 1200, RRID: SCR_018453). Free‐floating sections were preincubated for 1 h with 5% normal goat serum (NGS; Dianova) in saline‐substituted Tris buffer (SST; 0.1 mol L^−1^ Tris–HCl/0.3 mol L^−1^ NaCl, pH 7.4) containing 0.5% Triton X‐100 (TrX). Sections were subsequently incubated overnight in primary antibody solution, consisting of anti‐LomTK‐II antiserum, diluted at 1:12,000 to 1:20,000, or anti‐LomTK‐I antiserum, diluted at 1:4000 to 1:8000, in SST containing 0.5% TrX and 1%–2% NGS. After thorough rinses in SST (0.1% TrX), sections were incubated in secondary antiserum, goat anti‐rabbit (1:40; Sigma, RRID: AB_261363), followed by thorough rinses, and the PAP complex (rabbit PAP, 1:300; Dako; RRID: AB_2315056), both diluted in SST, 0.5% TrX, and 2% NGS and incubated at room temperature for 1 h each. The sections were rinsed and finally treated with 3,3′‐diaminobenzidine tetrahydrochloride (0.33 mg mL^−1^, Sigma Aldrich) and 0.05% H_2_O_2_ in sodium phosphate buffer (0.1 mol L^−1^, pH 7.4) for 10–30 min until a dark‐brown reaction product appeared. In some experiments the staining was intensified and converted into a dark blue reaction product by the addition of 0.3% nickel ammonium sulfate as described by (Loesel and Homberg 1999). Staining was stopped by rinses in sodium phosphate buffer. The sections were mounted on chrome alum/gelatin‐coated slides, dehydrated, cleared, and embedded in Entellan (Merck) using coverslips.

For immunofluorescence labeling of whole mount brains, fixed brains were rinsed in PBS (0.01 mol L^−1^, pH 7.4) containing 0.3% TrX and preincubated overnight in 5% NGS in PBS with 0.3% TrX. The following day, the brains were incubated in anti‐LomTK‐I antiserum (1:4000) or anti‐LomTK‐II antiserum (1:10,000–1:12,000), in some experiments combined with monoclonal anti‐synapsin antibody (1:100) diluted in PBS, 0.3% TrX, and 2% NGS for 2–4 days at 4°C. After washing in PBS/0.3% TrX for 5 × 10 min, brains were incubated for 2–3 days in Cy3‐conjugated goat anti‐rabbit (1:300, Jackson ImmunoResearch, cat# 111‐165‐144, RRID: AB_2338006) and, for detecting synapsin, Cy5‐conjugated goat anti‐mouse (1:300, Jackson ImmunoResearch, cat# 115‐175‐146, RRID: AB_2338713), diluted in PBS with 0.3% TrX and 2% NGS. Following thorough rinses in PBS, brains were either embedded in Mowiol (Mowiol Embedding Medium 2010) or, after dehydration through an ascending series of ethanol solutions and clearing in methyl salicylate, in Permount (Fisher Scientific).

### 2.3 Characterization of Antibodies

The antiserum against LomTK‐I (#9207‐7; RRID: AB_3675603) was a gift from Dr. Dick Nässel (Stockholm University). The antiserum was raised in rabbit against LomTK‐I (GPSGFYGVR‐NH_2_) conjugated to human serum albumin via carbodiimide (Nässel 1993). The antiserum has been used to study the distribution of tachykinin‐related peptides in the brains of the migratory locust (Nässel 1993), the blowfly *Calliphora vomitoria* (Lundquist et al. 1994), and the cockroach *R. maderae* (Muren et al. 1995). On brain sections of the cockroach *R. maderae* and the locust *S. gregaria*, the patterns of LomTK‐I and Lom‐TK‐II immunostaining were indistinguishable (Muren et al. 1995; Vitzthum and Homberg 1998). Preadsorption of the diluted LomTK‐I antiserum with 20 nmol mL^−1^ LomTK‐I or LomTK‐II abolished all immunostaining in the brains of the migratory locust, blowfly, and cockroach (Nässel 1993; Lundquist et al. 1994; Muren et al. 1995), whereas the preimmune serum did not produce any immunolabeling.

The antiserum against LomTK‐II (K1‐50820091; RRID: AB_2341129) was a gift from Dr. Hans Agricola (University of Jena, Germany). It was raised in rabbit against LomTK‐II (APLSGFYGVR‐NH_2_), coupled to bovine thyroglobulin via glutaraldehyde (Veenstra et al. 1995). The antiserum has been used to map LomTK‐II immunolabeled neurons in the gut and brain of various insect species, including the desert locust (Vitzthum and Homberg 1998), mealworm (Wegerhoff et al. 1996), coleopterans (Kollmann et al. 2016; Binzer et al. 2014), tobacco budworm (Berg et al. 2007), honeybee (Kaiser et al. 2022), mosquito (Veenstra et al. 1995), the dipluran *C. augens* (Böhm et al. 2012), and the crayfish *Cherax destructor* (Utting et al. 2000). An enzyme‐linked immunosorbent assay (ELISA) showed that the antiserum fully crossreacts with LomTK‐I as well as with CavTK‐I and II, two tachykinins from the blowfly *C. vomitoria* (Nässel et al. 1995). The specificity of the antiserum was further characterized in preadsorption controls on brains of the desert locust (Vitzthum and Homberg 1998), the mealworm beetle (Wegerhoff et al. 1996), and the red flour beetle (Binzer et al. 2014) and on mosquito midgut (Veenstra et al. 1995). Liquid‐phase preadsorption of the diluted antiserum with 100 µmol L^−1^ GABA‐glutaraldehyde conjugate, leucokinin, or substance P did not reduce immunostaining, whereas preadsorption with 10 µmol L^−1^ synthetic LomTK‐II abolished all immunostaining in brain sections of *S. gregaria* (Vitzthum and Homberg 1998). Likewise, preadsorption of the diluted antiserum with 20 µmol L^−1^ LomTK‐II abolished immunostaining on brain sections of the mealworm beetle *Tenebrio molitor* (Wegerhoff et al. 1996). Finally, preadsorption of the diluted antiserum with 10 µmol L^−1^ abolished immunostaining in the brain of the red flour beetle *T. castaneum* (Binzer et al. 2014) and in the midgut of the mosquito *Aedes aegypti* (Veenstra et al. 1995).

The monoclonal antibody against synapsin (#3C11, RRID: AB_2315425) was raised in mouse against parts of the *Drosophila* synaptic vesicle protein SYN1 fused with glutathione‐*S*‐transferase (Klagges et al. 1996). Its specificity has been demonstrated in *Drosophila* by Klagges et al. (1996). The antibody has been used to label synaptic neuropils in various insect species, including the desert locust (Kurylas et al. 2008; von Hadeln et al. 2020), honeybee (Brandt et al. 2005), Madeira cockroach (Althaus et al. 2022), dung beetle (Immonen et al. 2016), and ants (Bressan et al. 2015; Habenstein et al. 2020).

### 2.4 Data Evaluation

Images from PAP‐labeled brain sections were captured by a digital camera (ProgRes C12plus, Jenoptik) mounted on a Zeiss Axiophot compound light microscope. Images were optimized in contrast and brightness using Photoshop 2021 (Adobe Systems; RRID: SCR_014199). Immunolabeled neurons were traced from the sections through a camera lucida attachment on a compound microscope (Leitz). Drawings were digitized with a scanner (CanoScan LiDE 400, Canon). Images and 2D‐reconstructions were assembled in Illustrator 2021 (Adobe Systems; RRID: SCR_010279).

Immunofluorescent whole mount preparations and sections were scanned by a confocal laser scanning microscope (TCS SP5 II, Leica Microsystems; RRID: SCR_018714). Synapsin labeling (Cy5) was excited by a helium–neon laser (633 nm) and LomTK immunofluorescence (Cy3) with a DPSS‐laser (561 nm) or, for detecting Cy2 fluorescence, with an argon‐laser (488 nm). Overview scans were obtained with a 20x oil immersion objective (HC PL APO 20x/0.75 LMM Corr CS2) using a speed of 200 Hz and a line average of 2. Detailed scans were obtained with a 40x oil immersion objective (HCX PL APO lambda blue 40x/1.25 oil UV) at a step size of 1 µm, a pixel size of 0.75 µm × 0.75 µm in the *xy*‐plane, and a scan frequency of 200 Hz, and a 63x oil immersion objective (HCX PL APO 63x/1.40 oil PH3) at a step size of 1 µm, a pixel size of 0.12 µm × 0.12 µm in the *xy*‐plane, and a scan frequency of 400 Hz. Image stacks were evaluated and merged with Amira 5.6 and 6.5 (Thermo Fisher Scientific; RRID: SCR_007353). For 2‐dimensional reconstructions, labeled neurons were traced digitally in Photoshop 2021 based on serial images. Photoshop 2021 and Illustrator 2021 were used to optimize contrast and brightness of images and to create the figure panels.

The terminology of brain areas follows the nomenclature of Ito et al. (2014); the classification of neuronal cell types is based on Heinze and Homberg (2008) and von Hadeln et al. (2020), and, for flies, on Hulse et al. (2021). Positional information is given with reference to the animal's body axis.

### 2.5 Sequence Analysis

Tachykinin sequences from the 25 species listed in Table 1 were obtained through an extensive literature survey and database mining using OrthoDB (https://www.orthodb.org) and NCBI (https://www.ncbi.nlm.nih.gov). For species lacking annotated tachykinin sequences, prepropeptides were identified from closely related taxa and used as queries for BLAST searches in NCBI transcriptome shotgun assembly (TSA) or sequence read archive (SRA) datasets.

## 3 Results

In total 25 species from 12 hexapod orders were studied. Immunostaining using the LomTK‐I and LomTK‐II antisera differed slightly in background labeling but otherwise resulted in identical staining patterns in the CX of a given species, as shown in the cockroach *R. maderae* (Muren et al. 1995; this study), the locust *S. gregaria* (Vitzthum and Homberg 1998), the damselfly *C. puella* (this study), the honeybee *A. mellifera* (Kaiser et al. 2022; this study), and the fruit fly *D. melanogaster* (this study). In all other species, either the LomTK‐I or the LomTK‐II antiserum was used. Differences in staining quality, such as distinctness of labeling and signal to background level, were observed between representatives of different orders, which prevented clear identification of the labeled cell types in some species, notably the two lepidopterans studied. In most orders, distinct sets of columnar neurons were immunolabeled. In contrast in Diptera, as well as additionally in certain other orders, tangential neurons showed LomTK immunoreactivity. No immunostaining was found in the brain of the cricket *A. domesticus*.

### 3.1 LomTK Immunostaining in the CX of a Two‐Pronged Bristletail (Diplura)

**FIGURE 1:**
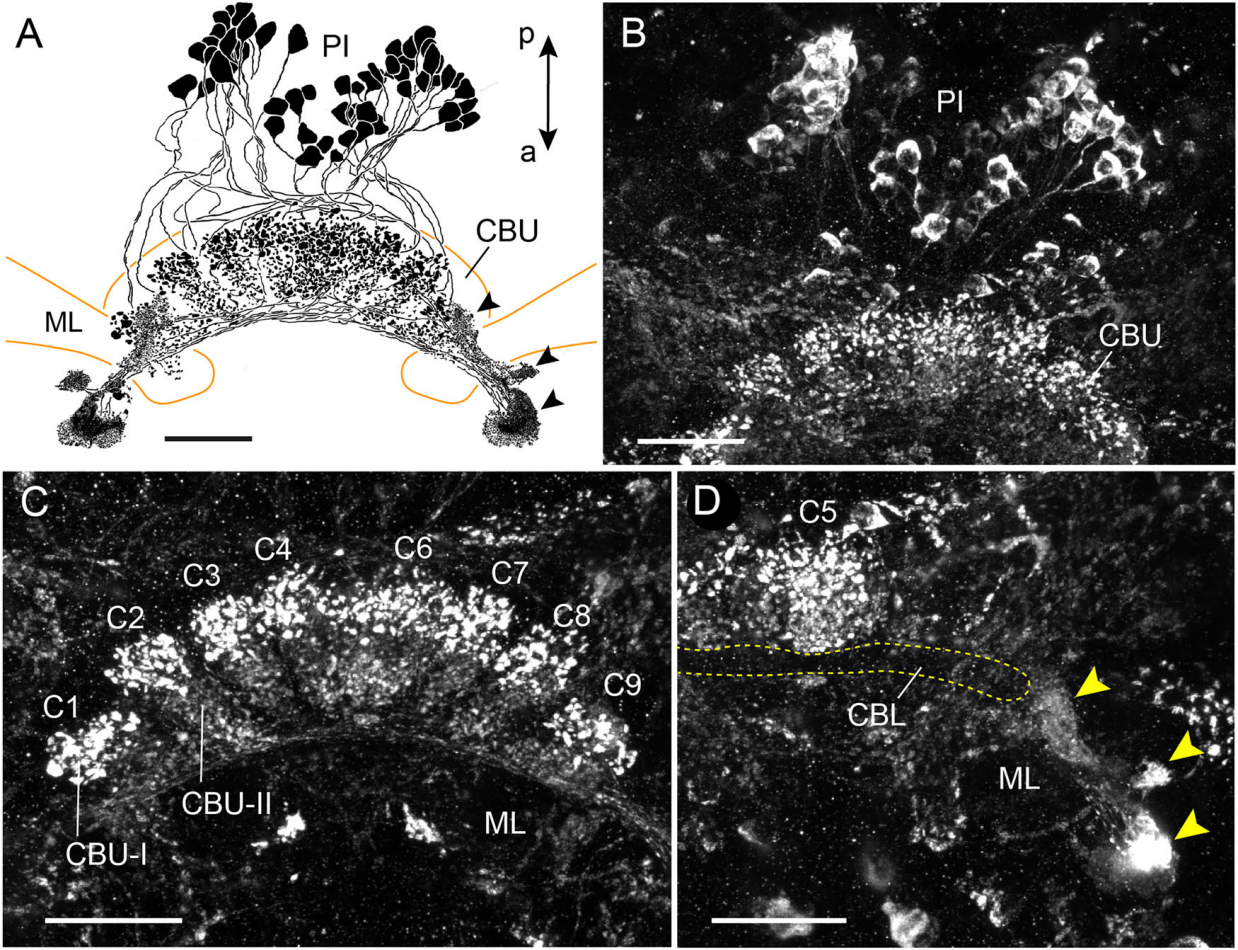
LomTK‐II immunostaining in the central complex of the two‐pronged bristletail *Campodea augens*. (A) Horizontal reconstruction (dorsal view) of the immunolabeled neurons. More than 50 neurons with somata in the pars intercerebralis (PI) innervate the upper division of the central body (CBU). Immunostaining in the CBU has a varicose appearance and is concentrated in nine columnar domains. Processes from the neurons continue to fine dense terminals in three areas (arrowheads) within or adjacent to the lateral complex. (B) Stack of optical sections showing immunolabeled cell bodies in the PI. (C) Stack of optical sections through the CBU. Varicose terminals are concentrated in nine columnar domains (C1–C9) and extend across two layers, a strongly labeled dorso‐posterior layer (CBU‐I) and a more sparsely invaded anterior layer (CBU‐II). (D) Stack of sections dorsal to that in (C) illustrating lack of immunostaining in the lower division of the central body (CBL), innervation of the dorsally protruding column C5 of the CBU, and fine dense terminals in three small areas (yellow arrowheads) in close neighborhood to the medial lobe of the mushroom body (ML). Double arrow in (A) indicates orientation (a, anterior; p, posterior) relative to body axis and applies to (A–D). Scale bars = 30 µm.

As a representative of Diplura, we studied LomTK‐II immunostaining in the CX of the two‐pronged bristletail *C. augens* (Figure 1). Our data confirm and extend the study of Böhm et al. (2012) on the organization of the CX of *C. augens*. The cerebrum of *C. augens* is tilted backwards relative to that of a locust or honeybee so that its anterior–posterior axis roughly corresponds to a dorsal–ventral axis in the locust and honeybee. About 53 neurons with somata in the pars intercerebralis exhibit LomTK‐II immunostaining (Figure 1A,B). Their cell body fibers enter the central body in several fascicles at its posterior face and give rise to dense varicose labeling in nine distinct columns of the CBU, four lateral columns on the right and four on the left (C1–C9 in Figure 1C), and a large midline‐spanning column C5 that protrudes dorsally from the others (Figure 1D). No staining was detected in the PB. Staining in the CBU columns is concentrated in a posterior–dorsal layer of the CBU (CBU‐I) and extends with less dense varicosities to an adjacent anterior layer (CBU‐II). No staining was detected in the CBL (Figure 1D). Fine neurites continue from the base of the columns anterior‐laterally along the ventral face of the medial lobes of the mushroom bodies and terminate in three small distinct areas that are densely supplied by fine processes (Figure 1A,D). Judged from this staining pattern, the labeled neurons are one or several types of CU columnar neurons of the CBU with processes extending to the crepine or rubus of the brain.

### 3.2 Immunostaining in the CX of the Firebrat (Zygentoma)

**FIGURE 2:**
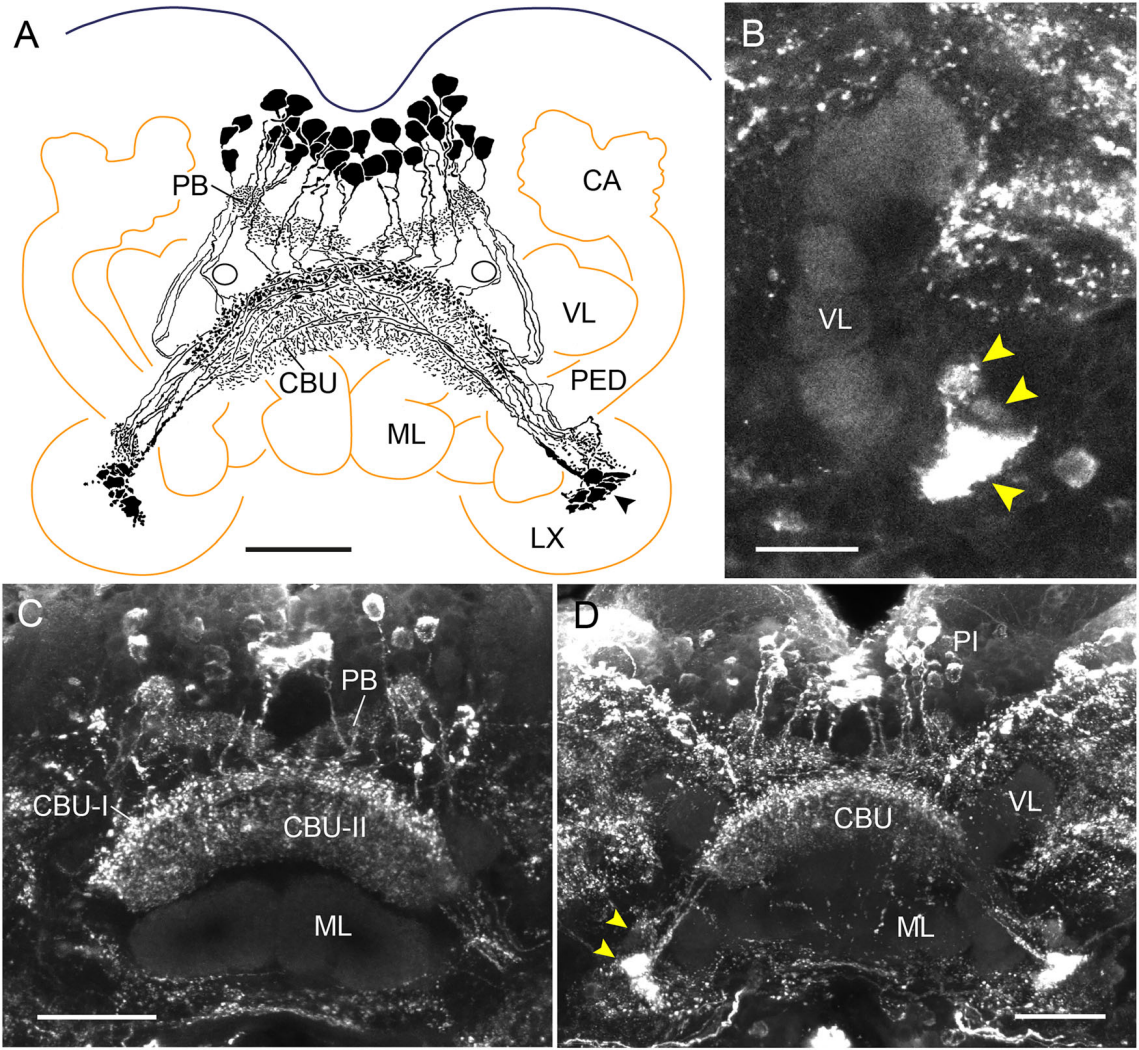
LomTK‐II immunostaining in the central complex of the firebrat *Thermobia domestica*. (A) Frontal reconstruction of immunolabeled neurons. About 33 neurons with cell bodies in the pars intercerebralis send neurites to the protocerebral bridge (PB), the upper division of the central body (CBU), and continuing processes to densely innervated small domains near the mushroom body lobes (arrowhead). Black circles indicate cross section of the medial antennal lobe tracts. (B) Stack of sections illustrating immunostaining in small domains laterally from the transition of the medial and vertical lobe (VL) of the mushroom body (yellow arrowheads) on the right side of the brain. (C) Stack of optical sections showing innervation of the PB and two layers of the CBU (CBU‐I and CBU‐II). (D) Stack of optical sections anterior to those in (C) showing labeled cell bodies in the pars intercerebralis (PI), staining in the CBU, and dense labeling of terminal processes of the neurons in small inferior neuropils (yellow arrowheads). Scale bars = 50 µm (A, C, D), 30 µm (B). CA, calyx of the mushroom body; LX, lateral complex; ML, medial lobe; PED, pedunculus.

As shown previously in the silverfish *Lepisma saccharina* using an antiserum against LomTK‐I (Loesel et al. 2002), the PB and the CBU exhibit tachykinin immunolabeling in the firebrat *T. domestica* (Figure 2). In total, a system of about 33 columnar neurons with somata in the pars intercerebralis, similar to the labeled neurons found in *C. augens*, shows LomTK‐II immunostaining. Cell body fibers invade the PB and continue in five bilateral pairs of fiber bundles to the CBU. Fibers especially from the three innermost bundles cross the brain midline in the posterior chiasma before entering the CBU, whereas fibers from the two outermost bundles pass laterally around the medial antennal lobe tract and innervate ipsilateral columns of the CBU. The neurons give rise to dense beaded staining in a dorsalmost layer of the CBU (CBU‐I), from where stained processes continue to less dense arborizations in a ventral layer of the CBU (CBU‐II, Figure 2A,C,D). From the anterior face of the CBU, main neurites project along the posterior face of the medial lobes of the mushroom body ventro‐laterally to three small adjacent brain areas (Figure 2A,B,D). Judged from the ramifications of the neurons in the PB, CBU, and small areas near the mushroom body lobes, the immunolabeled cells are CPU‐type neurons. The CBL is free of staining.

### 3.3 LomTK‐II Immunostaining in Dragonflies (Odonata)

**FIGURE 3:**
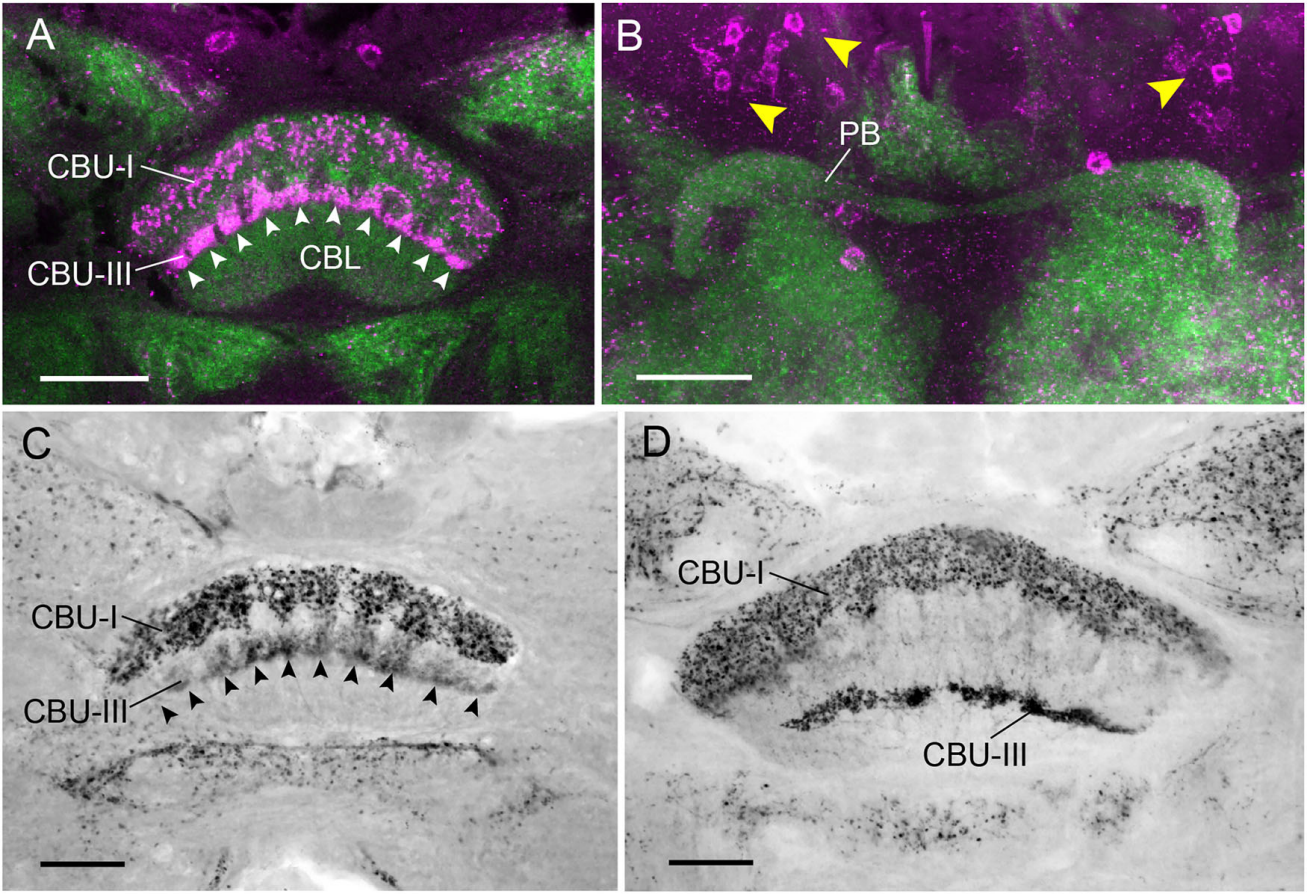
LomTK‐II immunostaining in the central complex of three species of Odonata. (A and B) *Platycnemis pennipes* (Zygoptera), (C) *Coenagrion puella* (Zygoptera), and (D) *Libellula quadrimaculata* (Anisoptera). (A) Single optical section, (B) stack of sections through the brain of *P. pennipes*. (C and D) Frontal peroxidase‐antiperoxidase labeled vibratome sections. In all three species, two layers in the upper division of the central body (CBU‐I and CBU‐III) exhibit LomTK‐II immunoreactivity (A, C, and D). In the two damselflies (A and C), immunostaining is concentrated in 10 columnar domains of the CBU, indicated by white (A), resp. black (C) arrowheads. (B) The protocerebral bridge (PB) of *P. pennipes* shows only sparse immunolabeling. Yellow arrowheads point at labeled cell bodies (magenta) in the posterior pars intercerebralis. Scale bars = 50 µm.

LomTK‐II immunostaining was studied in three species, the damselflies (Zygoptera) *P. pennipes* (Platycnemididae) and *C. puella* (Coenagrionidae) and the dragonfly *L. quadrimaculata* (Anisoptera, Libellulidae). In all three species, two layers of the CBU exhibit LomTK‐II immunolabeling, a dorsal layer (CBU‐I), which is stained in large varicosities, followed ventrally by an immunonegative layer (CBU‐II) and a most ventral layer (CBU‐III) which borders the CBL and shows dense LomTK‐II staining of fine appearance (Figure 3A,C,D). In the two damselfly species, immunostaining is concentrated in 10 columnar domains indicated by arrowheads in Figure 3A,C. The cellular origin of immunostaining in the CBU could not be uncovered as no fibers entering or leaving the CBU were labeled. The PB shows sparse staining in *P. pennipes* (Figure 3B) but was free of immunolabeling in *C. puella* and *L. quadrimaculata*. In contrast to labeling in the two‐pronged bristletail and firebrat, no immunostaining was present in defined areas of the crepine or lateral complex. Numerous scattered cell bodies are labeled in the pars intercerebralis (Figure 3B). Taken together, the staining pattern suggests that a particular type of ventral pontine neuron, corresponding to POUv neurons in the cockroach (Jahn et al. 2023) and vΔ neurons in *D. melanogaster* (Hulse et al. 2021), may be the cellular substrate of immunostaining in the CX. The noduli and CBL are devoid of labeling in all three species.

### 3.4 Immunostaining in the CX of Grasshoppers and Crickets (Orthoptera)

In Caelifera, LomTK immunostaining has been studied in the brain of the migratory locust *Locusta migratoria* (Nässel 1993) and in the CX of the desert locust *S. gregaria* (Vitzthum and Homberg 1998). The identification of immunolabeled neurons in the CX of the desert locust has been refined recently by double label experiments using antisera against myoinhibitory peptide (Hensgen, England, et al. 2021). In *S. gregaria* a particularly rich repertoire of cell types express tachykinin‐related peptides, and, accordingly, all neuropils of the CX except the noduli show LomTK‐II immunostaining. At least three types of columnar neuron and two types of tangential neuron show LomTK immunoreactivity.

**FIGURE 4:**
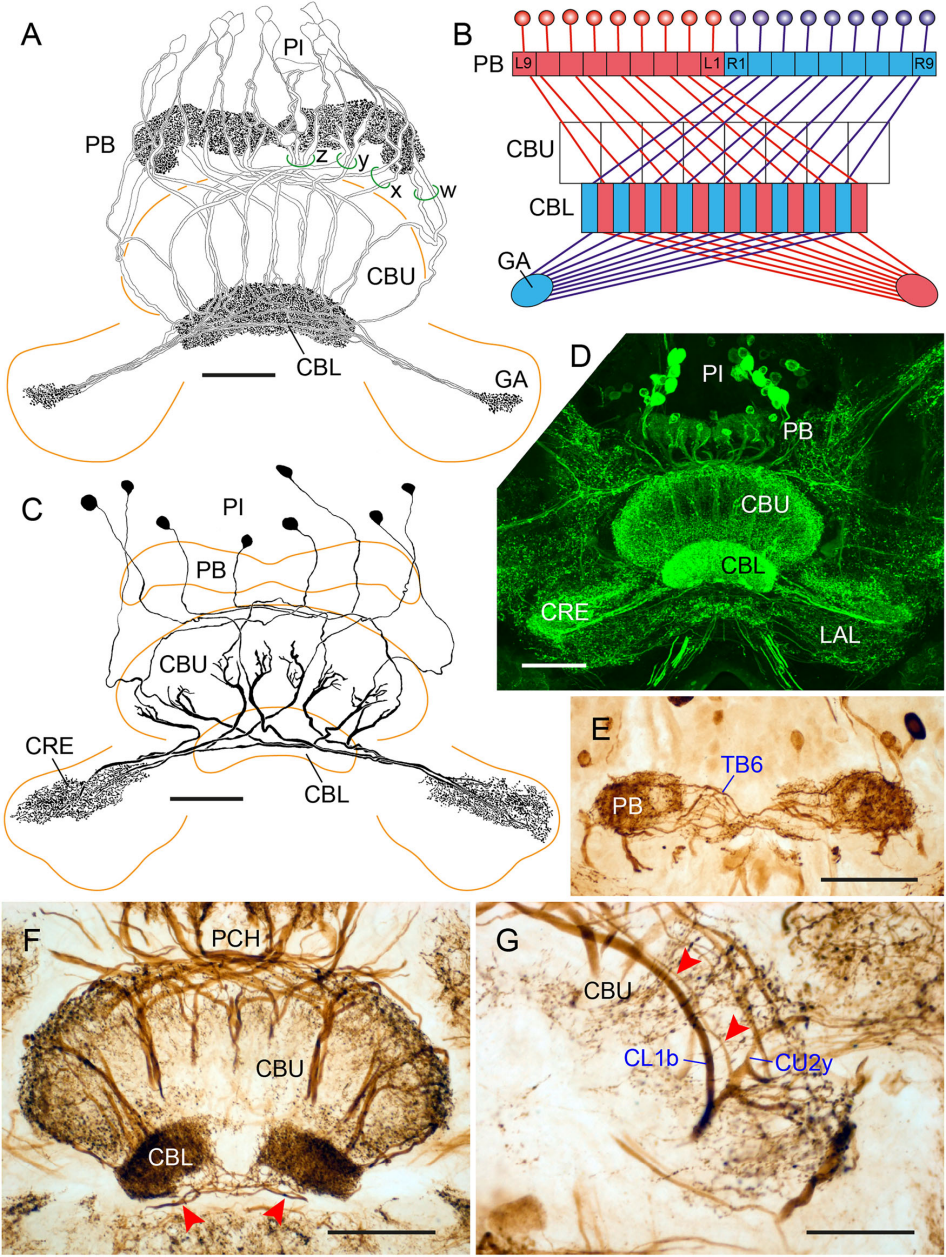
LomTK‐II immunostaining in the central complex of the desert locust, *Schistocerca gregaria*. (A) Frontal reconstruction of a system of 18 LomTK‐II‐immunolabeled CL1‐type columnar neurons with cell bodies in the pars intercerebralis (PI). The neurons give rise to granular staining in the protocerebral bridge (PB). Their main fibers project via the *w*‐, *x*‐, *y*‐ (two fibers each), and *z*‐bundles (three fibers) of the posterior chiasma to ramifications in the lower division of the central body (CBL). Axons continue via the contralateral isthmus tract (IT) and innervate the gall (GA) with beaded terminals. (B) Schematic wiring diagram of the immunolabeled CL1 neurons. L1, L9, column 1 resp. 9 of the left hemisphere of the PB; R1, R9, column 1 resp. 9 of the right hemisphere of the PB. (C) Frontal reconstruction of a system of eight immunolabeled CU2*x* neurons. Their cell‐body fibers project from the PI through the posterior chiasma to ramifications in the upper division of the central body (CBU). Axonal fibers continue along the anterior face of the central body to beaded arborizations in the contralateral crepine (CRE). (D) Stack of frontal optical sections showing LomTK‐II immunofluorescence in the central complex. (E) Frontal section showing immunostaining in the PB. Fibers of TB6 tangential neurons cross the midline of the PB. (F) Vibratome section showing immunoperoxidase labeling of LomTK‐II in the central body. Red arrowheads point at processes of two bilateral TL2 neurons innervating the CBL. (G) Detail from a frontal vibratome section showing ramifications of a CU2y neuron and its cell body fiber (red arrowheads) together with the main fiber of a CL1b neuron in the CBU. Scale bars = 100 µm (A–F), 50 µm (G). LAL, lateral accessory lobe.

Sixty to eighty cell bodies are immunolabeled in the pars intercerebralis (Figure 4) and most of these belong to neurons innervating the CX. Most prominently, 18 large CL1‐type columnar neurons, probably subtype CL1b (Hensgen et al. 2022), exhibit LomTK immunoreactivity (Figure 4A,B). The neurons have large, strongly stained cell bodies in the pars intercerebralis and innervate the PB. Large‐diameter fibers pass through the posterior chiasma with two fibers in *w*‐, *x*‐, and *y*‐bundles and three fibers in the *z*‐bundle to the central body (Figure 4A). The fibers traverse the CBU, give rise to dense ramifications in the CBL, and send continuing small axonal processes to the gall (Figure 4A). In addition, at least two systems of CU2 neurons, termed CU2x and CU2y (Hensgen et al. 2022), are immunolabeled. They correspond to LTC IV and LTC III neurons of Vitzthum and Homberg (1998). CU2x/LTC IV neurons, consisting of eight individual cells, have cell bodies in the pars intercerebralis (Figure 4C). Cell body fibers bypass the PB anteriorly, project via the posterior chiasma to the CBU, and join large‐diameter fibers that ramify in layers Ia and IIa of the CBU. Axonal processes with beaded terminals innervate the crepine (Figure 4C). A similar system of eight CU2y/LTC III neurons innervates layer Ib of the CBU (Figure 4G). The main fibers of these neurons accompany the neurites of the CL1b neurons through the CBU (Figure 4G). Like CU2x neurons, their axons give rise to beaded ramifications in the crepine. The identity of a fourth CL1 satellite system of columnar neurons with small fiber diameters, termed LTC II by Vitzthum and Homberg (1998), remains hypothetical.

In addition to columnar neurons, two types of tangential neuron exhibit LomTK immunostaining (Vitzthum and Homberg 1998). Three bilateral pairs of TB6 tangential neurons (von Hadeln et al. 2020), termed LTT II by Vitzthum and Homberg (1998), innervate the PB (Figure 4E). The neurons have wide ramifications in the posterior slope and give rise to beaded processes throughout the PB. Furthermore, two bilateral pairs of LomTK‐immunolabeled TL2 neurons, termed LTT I, innervate the lateral bulb and CBL (Figure 4F) as described by Vitzthum and Homberg (1998).

As a representative of the Ensifera we studied immunostaining in the brain of the house cricket *A. domesticus*. No immunolabeling was found in the CX as well as in the entire brain, suggesting that *A. domesticus* lacks tachykinin‐related neuropeptides. This result is supported by a recent peptidomics study on the field cricket *Gryllus bimaculatus*, which revealed complete absence of tachykinins (Mochizuki et al. 2022). Whether tachykinins are missing in all Gryllidae remains to be seen.

### 3.5 Immunostaining in the CX of a Stick Insect (Phasmatodea)

**FIGURE 5:**
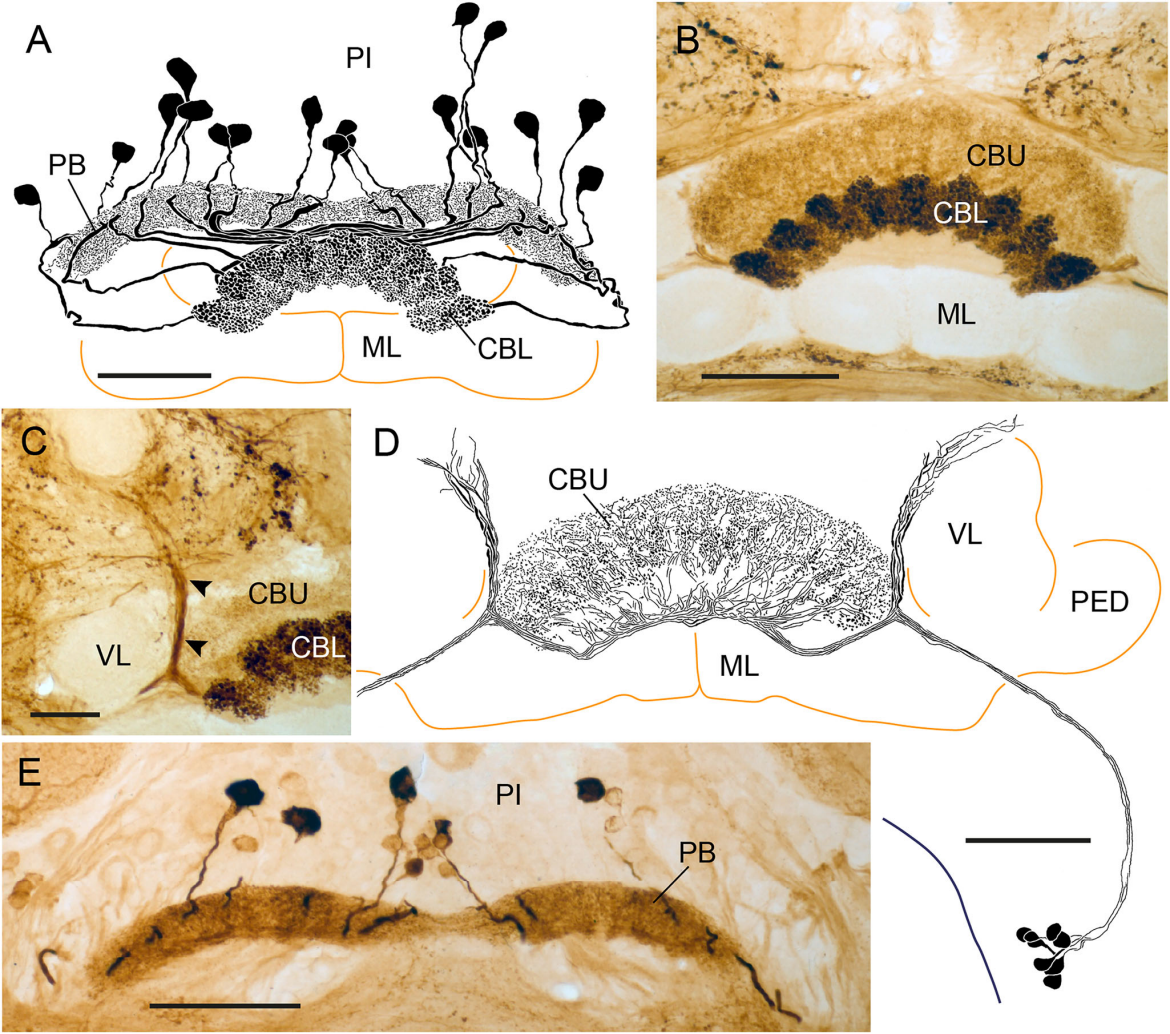
LomTK‐II immunostaining in the central complex of the Annam walking stick (*Medauroidea extradentata*). (A) Frontal reconstruction of a system of 18 CL1c‐type columnar neurons with cell bodies in the pars intercerebralis (PI). Their cell body fibers give rise to fine arborizations in the protocerebral bridge (PB). Neurites continue to the lower division of the central body (CBL) forming large, dense beaded terminals organized in 9 cone‐like domains. (B) Frontal vibratome section illustrating immunolabeling in the CBL and the upper division of the central body (CBU). (C) Neurites of immunolabeled TU_VES_4‐type neurons (arrowheads) pass along the inner margin of the vertical lobe (VL) of the mushroom body toward superior brain areas. (D) Frontal reconstruction of the TU_VES_ neurons. Their cell bodies are clustered near the vest (only shown in right brain hemisphere). The neurons innervate superior layers of the CBU with finely beaded processes. (E) Frontal vibratome section showing innervation of the PB by CL1c columnar neurons. Scale bars = 100 µm (A, B, D, E), 50 µm (C). ML, medial lobe; PED, pedunculus.

The central complex of the Annam walking stick (*M. extradentata*) is innervated by two types of LomTK‐II immunolabeled neuron, a system of 18 CL1c‐type columnar neurons, and a small group of TU_ves_‐type tangential neurons. Cell bodies of the columnar neurons are in the pars intercerebralis. The neurons send cell‐body fibers into the PB, which shows fine fibrous and partly beaded immunostaining (Figure 5A,E). Major neurites continue through the posterior chiasma of the central complex. The *w*‐, *x*‐, and *z*‐bundles of the chiasma each contain two neurites and the *y*‐bundle, three fibers. Neurites continue through the CBU and give rise to strongly immunolabeled large beaded terminals in nine cone‐like domains of the CBL and slightly smaller beads in the intercone domains (Figure 5A,B). Like CL1c neurons in the desert locust (Heinze and Homberg 2008) and dragonfly (Homberg, Kirchner, et al. 2023), the neurons lack axonal processes to the lateral complex.

In addition, bilateral groups of 7–8 TU_VES_4‐type tangential neurons innervating the CBU show tachykinin immunostaining (Figure 5C,D). The neurons have cell bodies clustered in the cell body rind medial from the vest. Cell body fibers form a tight bundle and approach the lateral edge of the central body in a wide arc without side branches. Here, their neurites bifurcate. Weakly stained processes project along the inner edge of the mushroom body's vertical lobe toward the superior brain but could not be traced to their final targets (Figure 5C,D). More strongly labeled neurites form a commissure along the posterior ventral edge of the CBL which is highly characteristic for TU_VES_4 neurons (von Hadeln et al. 2020; Vitzthum et al. 1996). Along that commissure, the neurons send numerous finely beaded processes into superior layers of the CBU (Figure 5D).

### 3.6 Immunostaining in the CX of Dictyoptera

**FIGURE 6:**
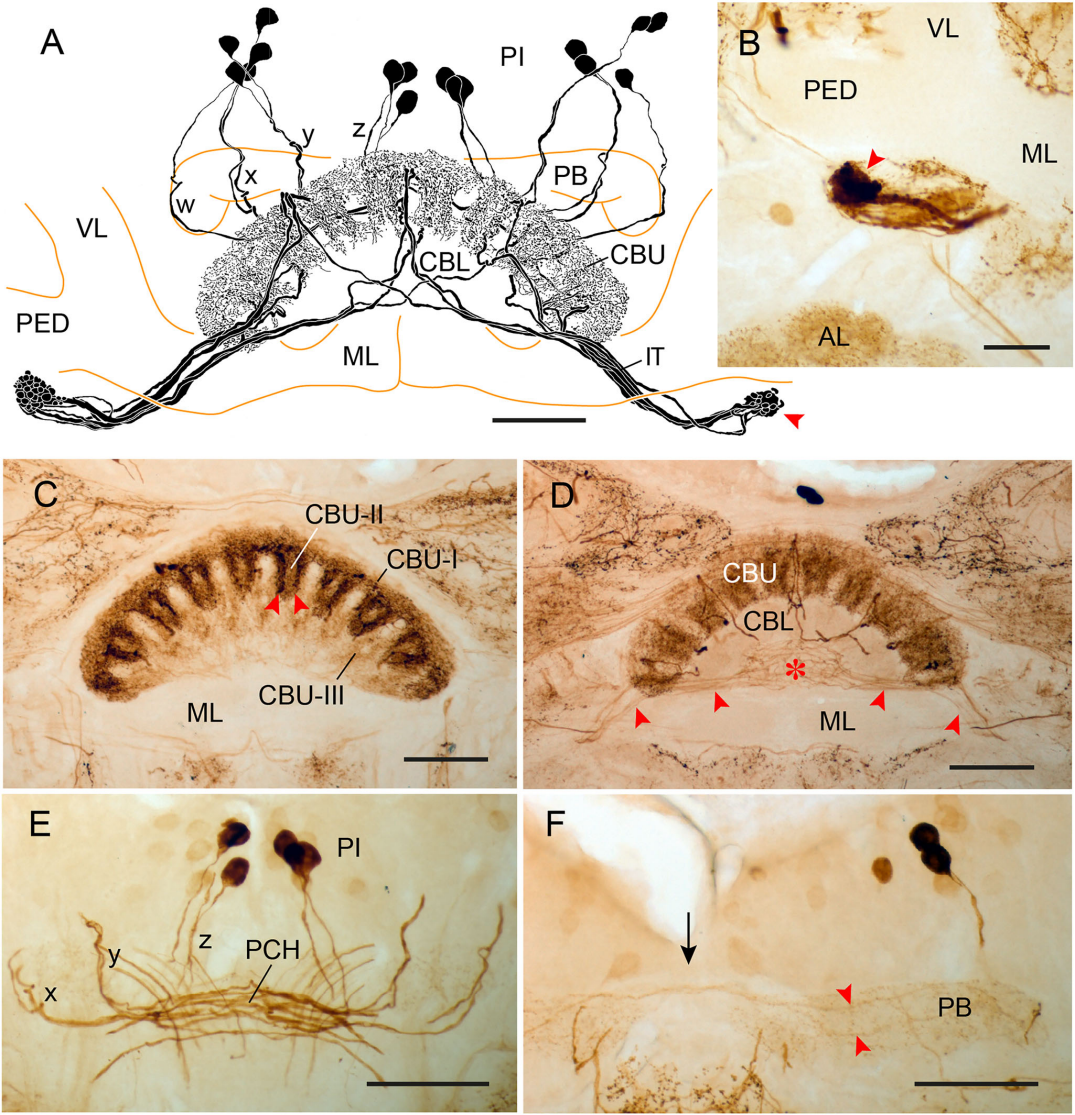
LomTK‐II immunostaining in the central complex of the praying mantis *Hierodula membranacea*. (A) Frontal reconstruction of a system of 16 immunolabeled CU2‐type columnar neurons with cell bodies in the pars intercerebralis (PI). Their cell body fibers give rise to mixed beaded/fine arborizations in the upper division of the central body (CBU). Axons project via the isthmus tracts (IT) to densely packed large knob‐like terminals in a small area near the pedunculus (red arrowhead). (B) Frontal vibratome section illustrating the small, densely invaded area below the pedunculus of the mushroom body, possibly corresponding to the rubus in the cockroach. (C and D) Frontal vibratome sections showing immunostaining in the CBU at a posterior (C) and more anterior (D) level. In the CBU, immunostaining of the CU2 neurons is concentrated in 10 columns of layer II (CBU‐II). Staining in the eight inner columns is further concentrated in two hemicolumns (red arrowheads in C). Weakly labeled fibers from tangential neurons (red arrowheads in D) give rise to a tangle of processes (red asterisk) in the space below the lower division of the central body (CBL). (E) Frontal vibratome section showing neurites of the CU2 neurons entering the posterior chiasma (PCH) via the *x*‐, *y*‐, and *z*‐bundles. (F) The protocerebral bridge (PB, only right hemisphere shown) is invaded by a bilateral pair of weakly stained tangential neurons (red arrowheads). Arrow indicates midline of the brain. Scale bars = 100 µm (A, C–F), 50 µm (B). AL, antennal lobe; ML, medial lobe; VL, vertical lobe of the mushroom body.

Immunostaining was studied in the praying mantis (*H. membranacea*) and the Madeira cockroach (*R. maderae*). In the mantis, a system of 16 columnar neurons innervating the CBU (CU2‐type neurons) was immunolabeled and, more weakly, a pair of tangential neurons of the PB (TB neurons) and a group of tangential neurons (TU neurons) innervating the CBU (Figure 6). Cell bodies of the CU neurons are in the pars intercerebralis (Figure 6A). Their cell body fibers bypass the PB anteriorly and enter the CBU via the posterior chiasma (Figure 6A,E). The *w*‐bundle contains one, the *x*‐ and *y*‐bundle two, and the *z*‐bundle, three immunolabeled neurites (Figure 6A). The neurons invade the dorsalmost layer I of the CBU, but mixed beaded/fine arborizations are particularly concentrated in 10 columnar domains of the underlying layer II (Figure 6C,D). They correspond to the 10 vertical slices reported by Rosner et al. (2017) and Althaus et al. (2024) based on synapsin labeling. Except for the right and left outermost columns, immunostaining in the eight inner columns is arranged in two semicolumns separated by less densely immunolabeled spaces (Figure 6C). Axonal fibers continue through the anterior chiasma into the contralateral isthmus tract and terminate in densely packed large knobs. These are concentrated in a small area ventrally from the pedunculi of the mushroom bodies (Figure 6A,B) likely corresponding to the rubus in the Madeira cockroach (Jahn et al. 2023). The connectivity matrix appears to follow that determined for CU2 neurons in the locust (Heinze and Homberg 2008). Two systems of tangential neurons show considerably weaker immunolabeling. The PB exhibits fine granular immunostaining originating from a bilateral pair of tangential neurons (TB neurons), but their ramifications and cell body positions outside the PB could not be identified (Figure 6F). Finally, layer III and perhaps the dorsalmost layer I of the CBU are uniformly invaded by processes from 6 to 8 bilateral pairs of small tangential neurons. Their neurites form a tangle of fibers in the space below the CBL (red asterisk in Figure 6D). Outside the CX, their fibers could only be traced for a short distance toward posterior dorsal brain areas where staining decreased to background levels.

**FIGURE 7:**
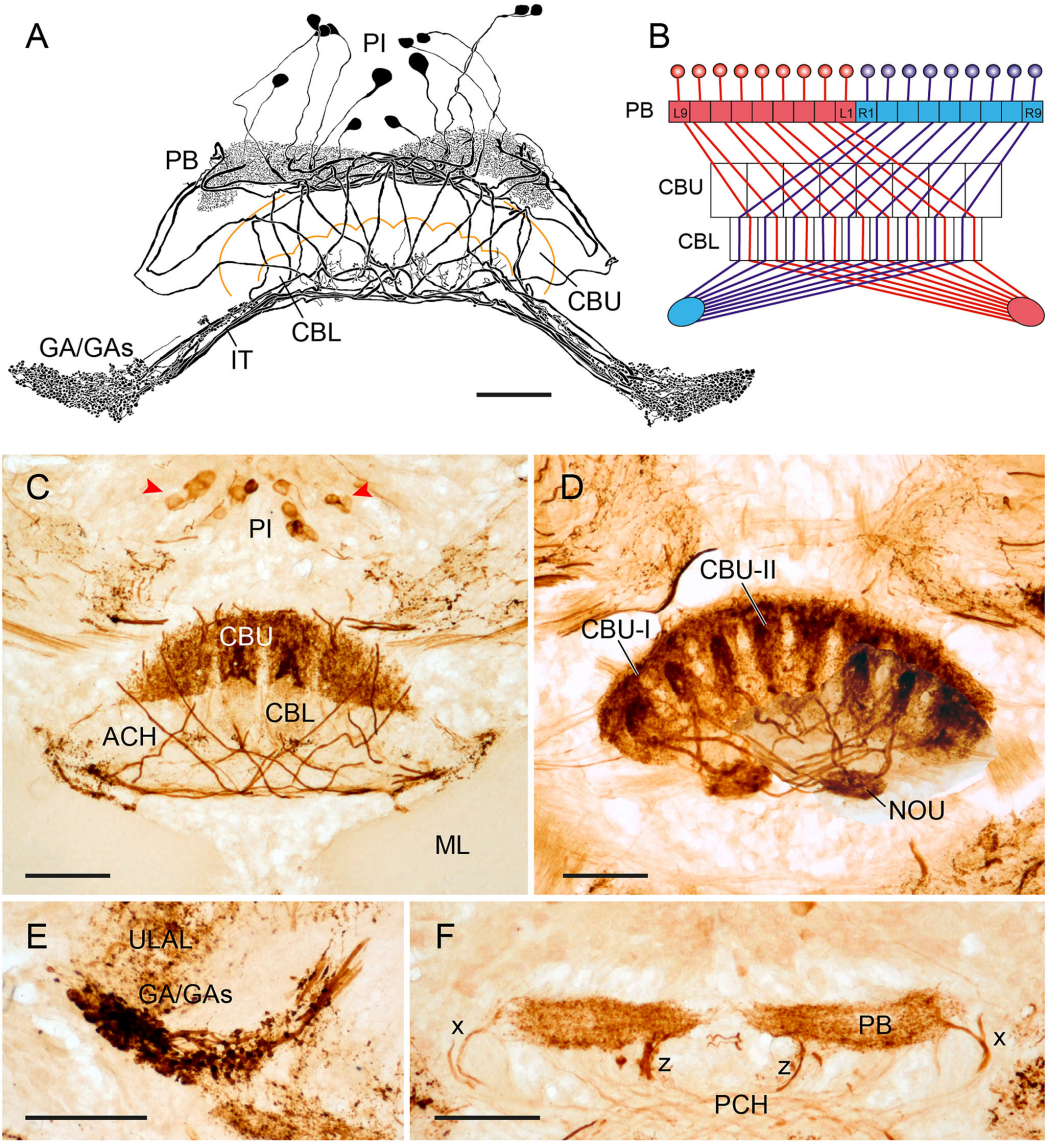
LomTK immunostaining in the central complex of the Madeira cockroach, *Rhyparobia maderae*. (C, E, and F) LomTK‐I immunostaining, (D) LomTK‐II immunostaining. (A) Frontal reconstruction of a system of 18 LomTK‐II‐immunolabeled CP2‐type columnar neurons with cell bodies in the pars intercerebralis (PI). Some cell bodies of CP2 neurons are obscured by the protocerebral bridge (PB). The neurons give rise to fine granular staining in the PB. Their main fibers project via the posterior and anterior chiasmata into the contralateral isthmus tract (IT) and innervate the gall (GA) and gall surround (GAs) with large beaded terminals. Sparse, finely beaded side branches extend from the main fibers into the lower division of the central body (CBL) and along the IT. (B) Schematic wiring diagram of the immunolabeled CP2 neurons. Side branches in the CBL are not shown for clarity. L1, L9, column 1 resp. 9 of the left hemisphere of the PB; R1, R9, column 1 resp. 9 of the right hemisphere of the PB. (C) Frontal vibratome section showing immunolabeled fibers of CP2 neurons in the anterior chiasma (ACH). Red arrowheads point to immunolabeled cell bodies of columnar neurons in the PI. (D) Image from two superimposed frontal sections through the CBL showing immunostaining in the upper division of the central body (CBU) and upper units of the noduli (NOU). Layer I of the CBU and eight columns in layer II of the CBU show dense immunostaining originating from immunolabeled CPU‐type columnar neurons connecting the CBU to the NOU. (E) Frontal section showing large beaded terminals of CP2 columnar neurons in the GA/GAs. (F) Frontal section showing immunostaining in the PB. Fibers of CP2 neurons in the *x*‐ and *z*‐bundles are visible. Scale bars = 100 µm. ML, medial lobe; ULAL, upper lateral accessory lobe.

In the cockroach *R. maderae* two systems of columnar neurons show LomTK immunostaining. One system consists of 18 CP2‐type columnar neurons connecting the columns of the PB to the gall and gall surround in the lateral complex (Figure 7A,B). From cell bodies in the pars intercerebralis, primary neurites give rise to fine ramifications in the PB (Figure 7A,F). Major fibers bypass the PB anteriorly. The neurites enter the posterior chiasma (two fibers each in the *w*‐, *x*‐, and *y*‐bundle, three fibers in *z*‐bundle) and pass along the anterior face of the CBU and CBL. Sparse beaded side branches are given off into the ventral base of the cones and teeth of CBL (Figure 7A,C). The main neurites enter the contralateral isthmus tract and give rise to prominently beaded terminals along the tract and more densely in the gall and gall surround (Figure 7E).

The second system of columnar neurons comprises 32 CPU neurons connecting the PB with layers I and II of the CBU and the noduli. Judged by comparison with single‐cell morphologies, the labeled neurons are most likely CPU5‐type columnar neurons of the CX (Jahn et al. 2023). From cell bodies in the pars intercerebralis, the neurons innervate the PB. Their main fibers continue through the posterior chiasma to the CBU. The neurons have mixed fine/beaded ramifications concentrated in eight columnar domains in layer II of the CBU and possibly also in layer I (Figure 7D). Large diameter neurites continue to layers II and/or III of the upper unit of the contralateral nodulus.

### 3.7 Immunostaining in the CX of Hemiptera

**FIGURE 8:**
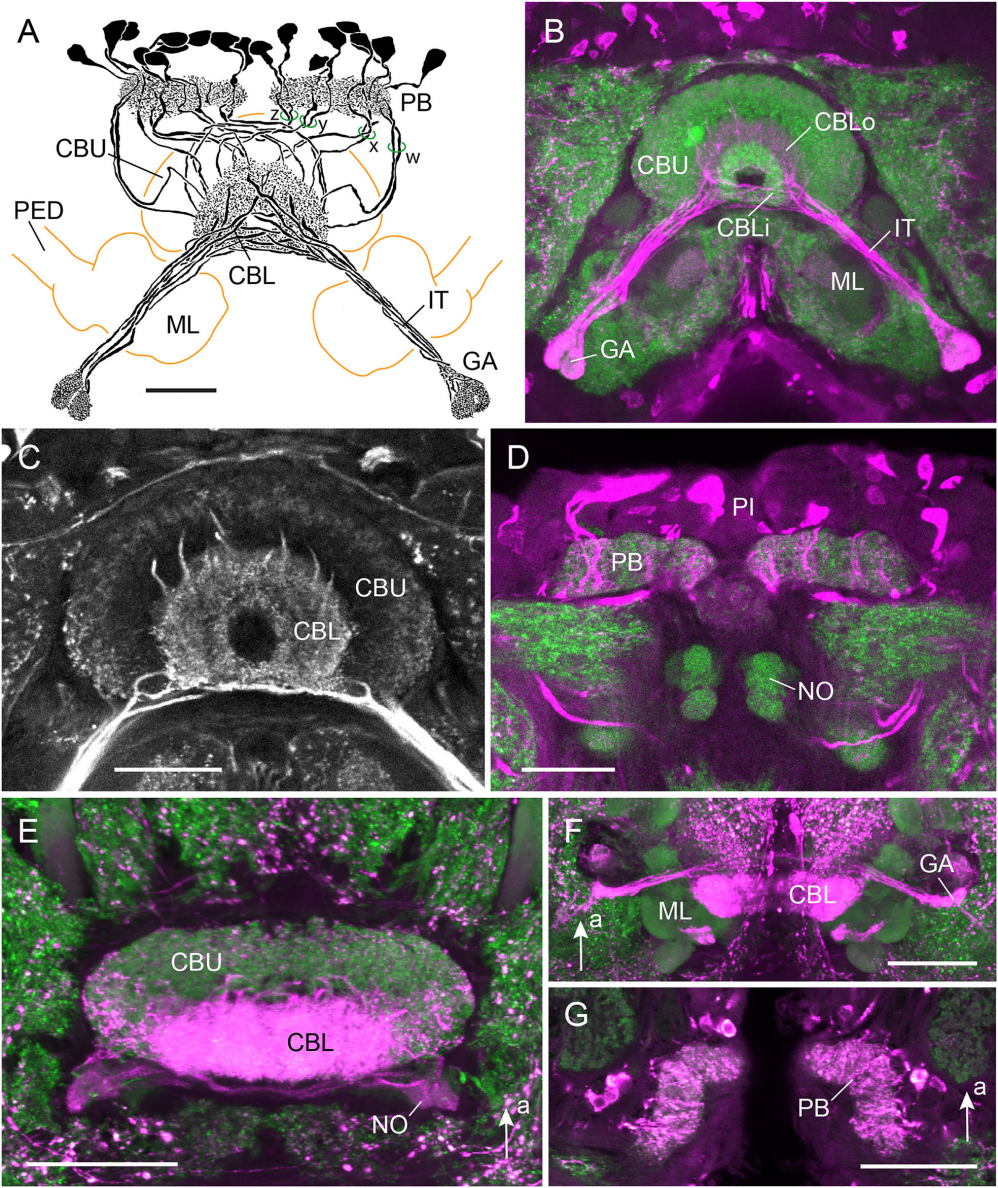
LomTK‐II immunostaining in the central complex of two hemipterans, the backswimmer *Notonecta glauca* (A–D) and the shield bug *Graphosoma italicum* (E–G). (A) Frontal reconstruction of a system of immunolabeled CL1 columnar neurons of the central complex. The neurons innervate the protocerebral bridge (PB) and, via the *w*‐, *x*‐, *y*‐, and *z*‐bundles, the lower division of the central body (CBL). Axons project via the isthmus tracts to densely packed terminals in the gall (GA). (B) Stack of five optical sections showing LomTK‐II immunolabeling (magenta) of the neurons in the CBL, isthmus tracts (IT), and GA. Based on differences in synapsin immunostaining (green), an inner (CBLi) and an outer (CBLo) layer of the CBL can be distinguished. The CBLi has a toroidal appearance. (C) Stack of optical sections illustrating LomTK immunostaining in the central body. (D) Stack of optical sections showing LomTK immunolabeling in the PB (magenta) and associated cell bodies in the pars intercerebralis (PI). (E–G) Shield bug, *Graphosoma italicum*, horizontal views. (E) LomTK immunolabeling (magenta) in the upper division of the central body (CBU), the CBL, and the noduli (NO). (F) Stack of optical sections illustrating immunolabeled fibers (magenta) projecting from the CBL to the GA. (G) Single optical section illustrating dense innervation of the PB by immunolabeled CL1 neurons. Scale bars = 50 µm. a, anterior; ML, medial lobe of the mushroom body; PED, pedunculus.

Immunostaining for LomTK was studied in the backswimmer *N. glauca* and in the shield bug *G. italicum* using the LomTK‐II antiserum (Figure 8). In both species the CBL/EB shows strongest immunoreactivity originating from systems of CL1‐type columnar neurons. In the backswimmer, the CBL has a toroidal structure, similar to the EB of the fruit fly (Figure 8B,C). Based on synapsin labeling, two layers can be distinguished in the CBL, an inner and an outer layer (Figure 8B). Only the inner layer forms the toroid, whereas the ends of the outer layer are not fused ventrally. Both layers are uniformly innervated by CL1 neurons. The neurons have large cell bodies in the pars intercerebralis, partly varicose ramifications in the PB, and send axonal fibers with densely stained terminals to the gall (Figure 8A–D). In *N. glauca*, two subdivisions of the gall could be distinguished (Figure 8A,B). Reconstruction of the CL1 neurons in one specimen of *N. glauca* revealed nine neurons in the right and 10 in the left hemisphere (Figure 8A). In the posterior chiasma, the *w*‐, *y*‐, and *z*‐bundles contained two fibers each and the *x*‐bundle three or four fibers. Owing to fusion of the columns in the CBL, the wiring scheme could not be elucidated but, judged from fiber trajectories, it likely corresponds to the scheme proposed for the locust (see Figure 4B). In both hemipterans, the CBU shows sparse beaded immunostaining in an outer layer (Figure 8B,C,E). In *N. glauca*, labeling of the CBU might originate from a second system of columnar neurons, because additionally labeled small cell bodies in the pars intercerebralis innervate the PB, but their fiber trajectories in the posterior chiasma and further course could not be revealed. In *G. italicum*, parts of the noduli show immunolabeling suggesting that CPU4 or CPU5 neurons exhibit LomTK immunostaining (Figure 8E).

### 3.8 Immunostaining in the CX of Hymenoptera

**FIGURE 9:**
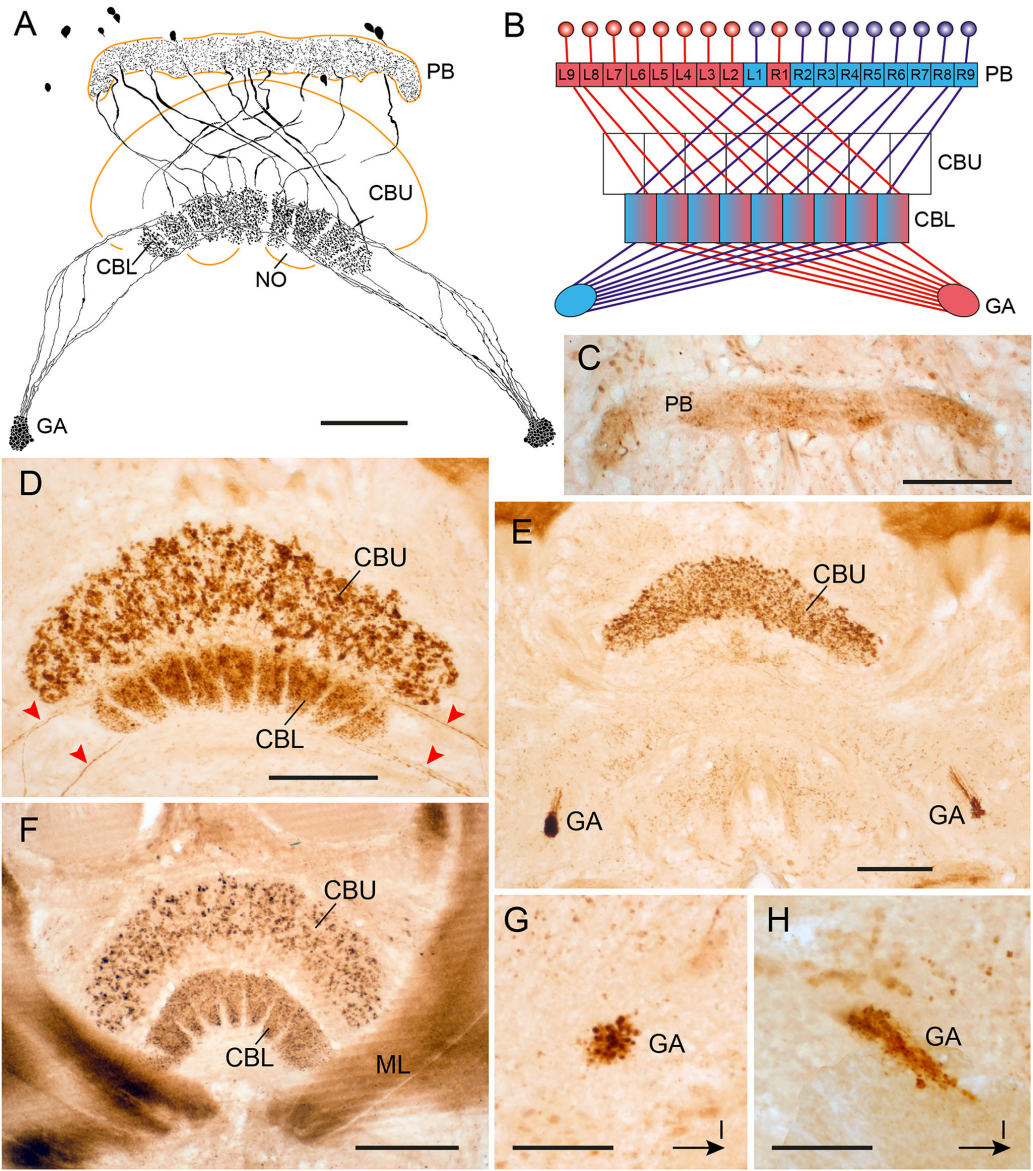
LomTK‐I immunostaining in the central complex of the German wasp *Vespula germanica* (A, C–E, G) and the honeybee *Apis mellifera* (F and H). (A) Frontal reconstruction of a system of immunolabeled CL1‐type columnar neurons of the central complex. The neurons innervate the protocerebral bridge (PB) and the lower division of the central body (CBL). Axons project via two fiber fascicles to densely packed beaded terminals in the gall (GA). (B) Proposed schematic wiring diagram of the immunolabeled neurons. The CBL and each hemisphere of the PB can be divided into nine columns. Except for neurons of the right and left innermost columns of the PB (L1, R1) all neurons connect PB columns to the contralateral gall (GA). (C) Frontal vibratome section showing immunostaining in the PB. (D) Frontal section through the central body. The upper division of the central body (CBU) shows immunostaining in large varicose terminals, whereas labeling in the CBL is of finer and denser appearance. Red arrowheads point at axonal processes in two bilateral fascicles targeting the GA. (E) At a more anterior level, axonal terminals in the right and left GA become visible. (F) Immunostaining in the central body of the honeybee is highly similar to the staining pattern in the wasp. (G and H) Higher magnification of CL1 terminals in the gall of the wasp (G) and honeybee (H, superimposed image from two adjacent sections). Scale bars = 100 µm (A–F), 50 µm (G and H). L, lateral; ML, medial lobe of the mushroom body.

Immunostaining for LomTK was studied in three hymenopteran species, the common wasp (*V. vulgaris*), the German wasp (*V. germanica*), and the honeybee (*A. mellifera*) using the LomTK‐I antiserum. In all three species, the CBU and CBL show immunoreactivity as described for the honeybee by Kaiser et al. (2022). The CBU shows varicose staining (Figure 9D–F), whereas immunostaining in the CBL is of a finer, punctate appearance and distributed in nine distinct columns (Figure 9D,F). The PB shows weak granular staining, and the NO are free of immunolabeling. Although the origin of immunoreactivity in the CBU could not be determined, immunostaining in the CBL originates from a system of CL1 neurons. In all species, a system of small fibers in the posterior chiasm and continuing axons to small areas in the lateral complex, apparently the gall, shows immunostaining, but cell bodies above the PB were not or only weakly labeled (Figure 9). In the German wasp, the CL1 fiber system could be partly reconstructed (Figure 9A). Staining in these neurons except for their terminals in the CBL and gall is weak and even absent in certain elements such as the fibers of the *w*‐bundles, suggesting low affinity of the hymenopteran tachykinins to the antisera. Reconstruction of the neurons is consistent with the projection pattern of CL1 neurons as reported for EPG/PEG neurons in the bumble bee (Sayre et al. 2021). The CBL and each hemisphere of the PB can be subdivided into nine columns (Figure 9B). Whereas neurons innervating the innermost PB columns L1 and R1 project to the ipsilateral gall, neurons of all other PB columns innervate the contralateral gall (Figure 9A,B). In the CBL, each of the nine columns is targeted by a neuron from the ipsi‐and contralateral PB column (Figure 9A,B). Axonal fibers project in two fascicles, one originating from the dorsal edge of the CBL and the other, from the ventral edge of the CBL toward the gall. Terminals in the spheroidal gall of the wasps have large beaded terminals (Figure 9G), whereas in the honeybee, the neurons have smaller irregularly beaded terminals forming an elongated gall (Figure 9H) as described for single CL1 neurons (Hensgen, England, et al. 2021).

### 3.9 Immunostaining in the CX of Coleoptera

**FIGURE 10:**
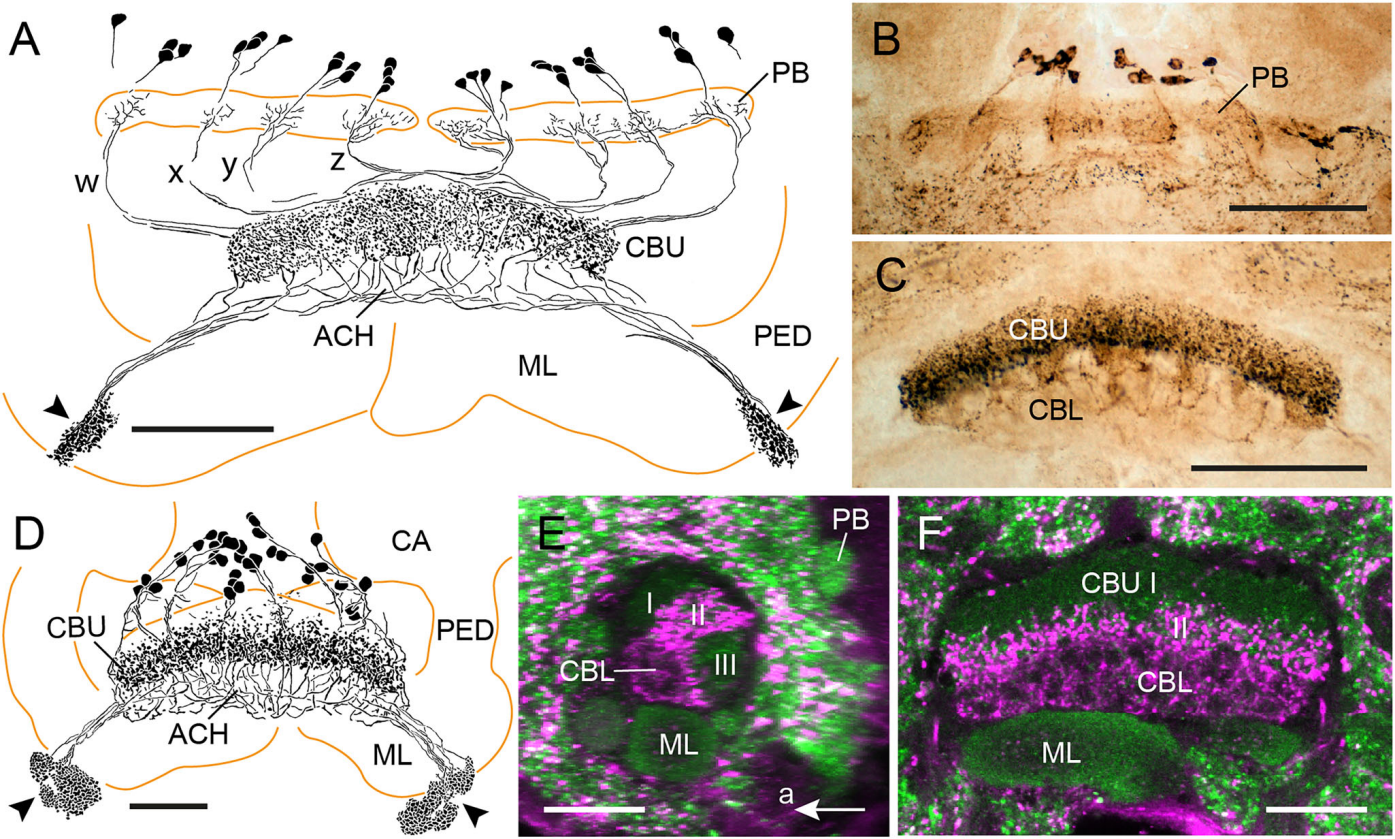
LomTK‐II immunostaining in the central complex of the darkling beetle *Zophobas morio* (A–C) and the red flour beetle *Tribolium castaneum* (D–F). (A and D) Frontal reconstructions of systems of 32 LomTK‐II immunolabeled CPU neurons, connecting the protocerebral bridge (PB) to the upper division of the central body (CPU) via four fiber bundles, the *w*‐, *x*‐, *y*‐, and *z* bundles. Axonal fibers continue via the anterior chiasma (ACH) to varicose terminals in small areas in the lateral complex (arrowheads). (B and C) Frontal vibratome sections illustrating LomTK immunostaining in the PB (B, superimposed image from two adjacent sections) and central body (C). (E) Sagittal projection of confocal images illustrating LomTK labeling (magenta) concentrated in the lower division of the central body (CBL) and in layer II (II) of the CBU. Synapsin staining is shown in green. (F) Single optical section showing immunostaining in the CBL and in layer II of the CBU (II). Scale bars = 100 µm (A–C), 20 µm (D and F). CA, calyx of the mushroom body; ML, medial lobe; PED, pedunculus.

Five coleopteran species were investigated, two species of Tenebrionidae (Polyphaga), the red flour beetle *T. castaneum* and the darkling beetle *Z. morio*, and three species of Dytiscidae (Adephaga), the diving beetles *A. sulcatus*, *G. substriatus*, and *I. fuliginosus*. In *T. castaneum* and *Z. morio*, systems of CPU columnar neurons exhibit dense immunoreactivity (Figure 10). In both species the CBU and small projection areas in the lateral complex showed most prominent varicose immunolabeling. Reconstruction of the neurons in *Z. morio* revealed eight clusters of neurons in the pars intercerebralis, each consisting of four neurons (Figure 10A,B). Their cell body fibers invade the PB, which is weakly labeled, and continue via the *w*‐, *x*‐, *y*‐, and *z*‐bundles to the CBU (Figure 10A–C). Axonal fibers project via the isthmus tracts to small areas of the lateral complex (arrowheads in Figure 10A), possibly corresponding to the gall or round body in *Drosophila*. A highly similar system of neurons, again consisting of 32 neurons in total, was found in *T. castaneum* (Figure 10D–F). In *T. castaneum*, the neurons invade an intermediate layer II of the CBU (Figure 10E). A corresponding system of LomTK‐immunolabeled neurons has been identified in the mealworm beetle *T. molitor* (Wegerhoff et al. 1996), however, consisting of 8 × 8 neurons instead of 8 × 4 as in *T. castaneum* and *Z. morio*. The identity of the immunolabeled CPU‐type neurons in *T. molitor* has been confirmed by singe‐cell dye injection into selected immunostained neurons (Wegerhoff and Breidbach 1994). In *Z. morio* and *T. castaneum*, the CBL shows faint immunostaining, but the labeled neurons could not be identified. No immunoreactivity was present in the NO.

**FIGURE 11:**
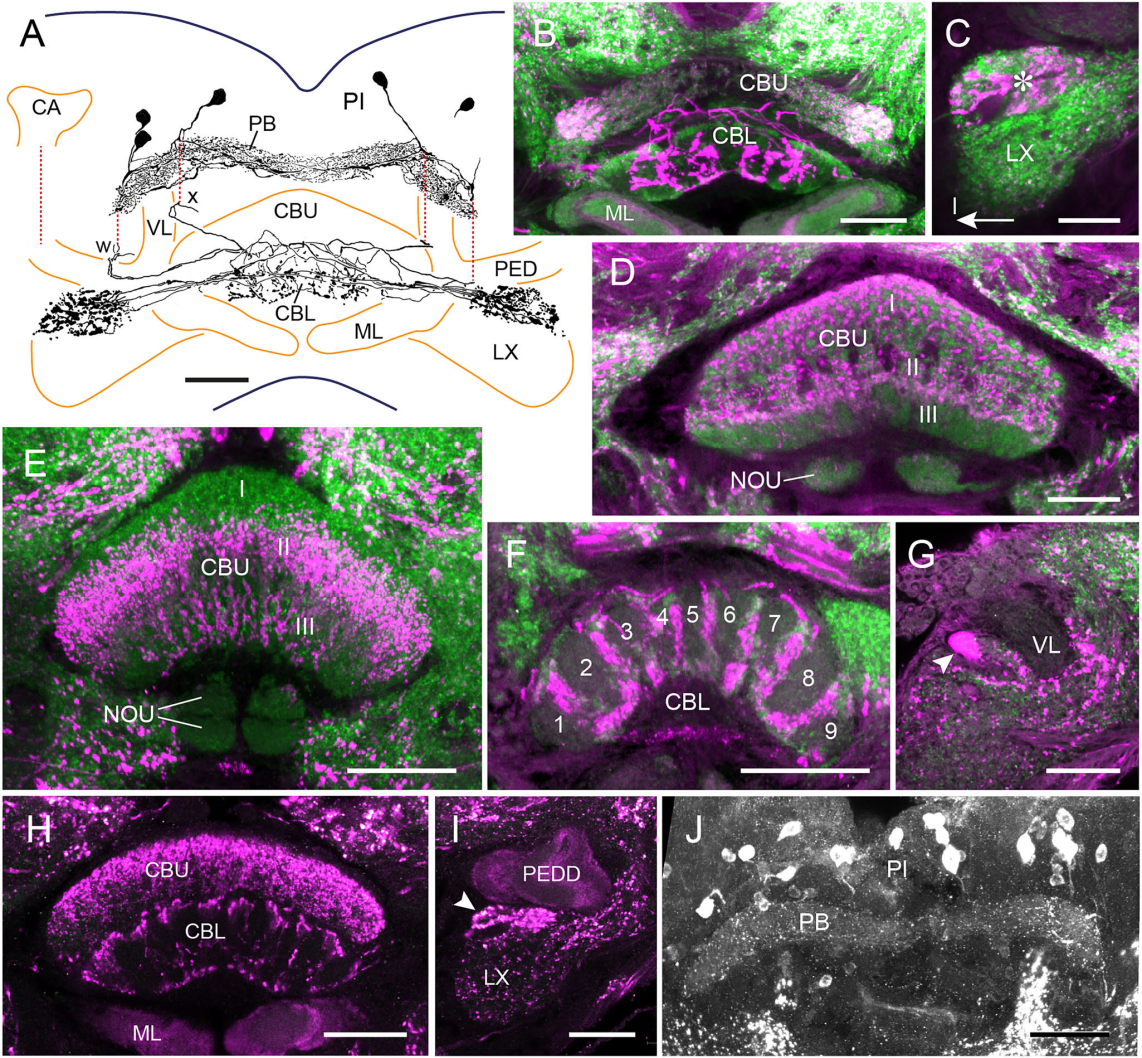
LomTK‐II immunostaining in the central complex of the diving beetles *Ilybius fuliginosus* (A–D)*, Gyrinus substriatus* (E–G), and *Acilius sulcatus* (H–J). (A) Frontal reconstruction of three bilateral pairs of LomTK‐II immunolabeled neurons with wide bilateral ramifications in the protocerebral bridge (PB) and large varicose terminals in the lower division of the central body (CBL) and areas in or near the lateral complex (LX). For clarity, ramifications in the PB are dorsally offset (red dotted lines). Two neurons project from the PB to the CBL via the *w*‐bundle (*w*), the third neuron, via the *x*‐bundle (*x*). (B–I) Single optical sections (C–E, G–I) and small stacks of sections (B, F, and J) from wholemount brains immunostained for LomTK‐II (magenta) and synapsin (green). (B) Stack of optical sections showing large varicose ramifications of LomTK‐II‐labeled neurons in the CBL. (C) The neurons have large varicose terminals in a small dorsal area (asterisk) in or adjacent to the lateral complex (LX). (D) Optical section through the upper division of the central body (CBU) showing different levels of beaded labeling in three layers (labeled I, II, and III) of the CBU. The upper units of the noduli (NOU) are sparsely invaded by immunoreactive profiles. (E) Optical section through the CBU of *G. substriatus*. A dorsal layer I lacks immunolabeled processes, whereas an intermediate layer II is densely and a ventral layer III more sparsely invaded by immunolabeled fibers. (F) Immunostaining in the CBL of *G. substriatus* is concentrated in synapsin‐rich areas dividing the CBL into nine columns (labeled 1–9). (G) Fibers originating from the CBL terminate in a small area (arrowhead) near the base of the vertical lobe (VL) of the mushroom body. (H) Single section through the central body of *A. sulcatus* showing dense beaded staining in a dorsal layer of the CBU and sparse labeling concentrated along the boundaries between columns of the CBL. (I) As in the other two species, fibers from the CBL extend to densely innervated bilateral areas in or adjacent to the lateral complex. (J) The PB of *A. sulcatus* exhibits only weak punctate labeling but numerous cell bodies in the pars intercerebralis (PI) above the PB are strongly labeled. Scale bars = 50 µm (A, E–J), 40 µm (B and D), 30 µm (C). CA, calyx (highly reduced); l, lateral; ML, medial lobe; PED, pedunculus.

The patterns of LomTK immunostaining are similar in the three species of diving beetle but differ considerably from staining in the tenebrionids. In all species the CBL is prominently invaded by immunolabeled neurons, which give rise to varicose ramifications concentrated along the boundaries between the columns (Figure 11A,B,F,H). In *I. fuliginosus* labeling in the CBL originates from three bilateral pairs of neurons (Figure 11A). The neurons have cell bodies in the pars intercerebralis. Their cell body fibers invade the PB with wide, apparently bilateral ramifications crossing the midline between the two halves of the PB. Neurites continue via the *w*‐ (two fibers) and *x*‐bundle (one fiber) through the posterior chiasma and invade the CBL with large varicose terminals. Each neuron sends axon collaterals bilaterally to varicose terminals in small areas in or near the lateral complex (Figure 10A,C). Similar neurons are labeled in *A. sulcatus* and *G. substriatus*, as judged by highly similar staining pattern in the CBL (Figure 11F,H) and continuing axons to densely innervated areas near the pedunculus (Figure 11G,I). In both species, however, stained fibers could not be traced to the PB, which, in contrast to *I. fuliginosus*, shows only weak immunostaining. In all three diving beetles, the CBU shows layer‐specific LomTK staining (Figure 11D,E,H), but the underlying neurons could not be identified. The large numbers of immunolabeled cell bodies in the pars intercerebralis (Figure 11J) with cell body fibers projecting toward the PB (6 strongly, 27 weakly labeled somata in *I. fuliginosus*; 4 large and 42 small somata in *G. substriatus*; 16 strongly, 52 weakly labeled somata in *A. sulcatus*) suggest that pontine neurons, perhaps corresponding to vΔ neurons in *D. melanogaster*, contribute to immunostaining in the CBU. The noduli are devoid of immunoreactivity or, as in *A. sulcatus* and *G. substriatus*, show weak immunostaining in their upper units (Figure 11D,E).

### 3.10 Immunostaining in the CX of Lepidoptera

**FIGURE 12:**
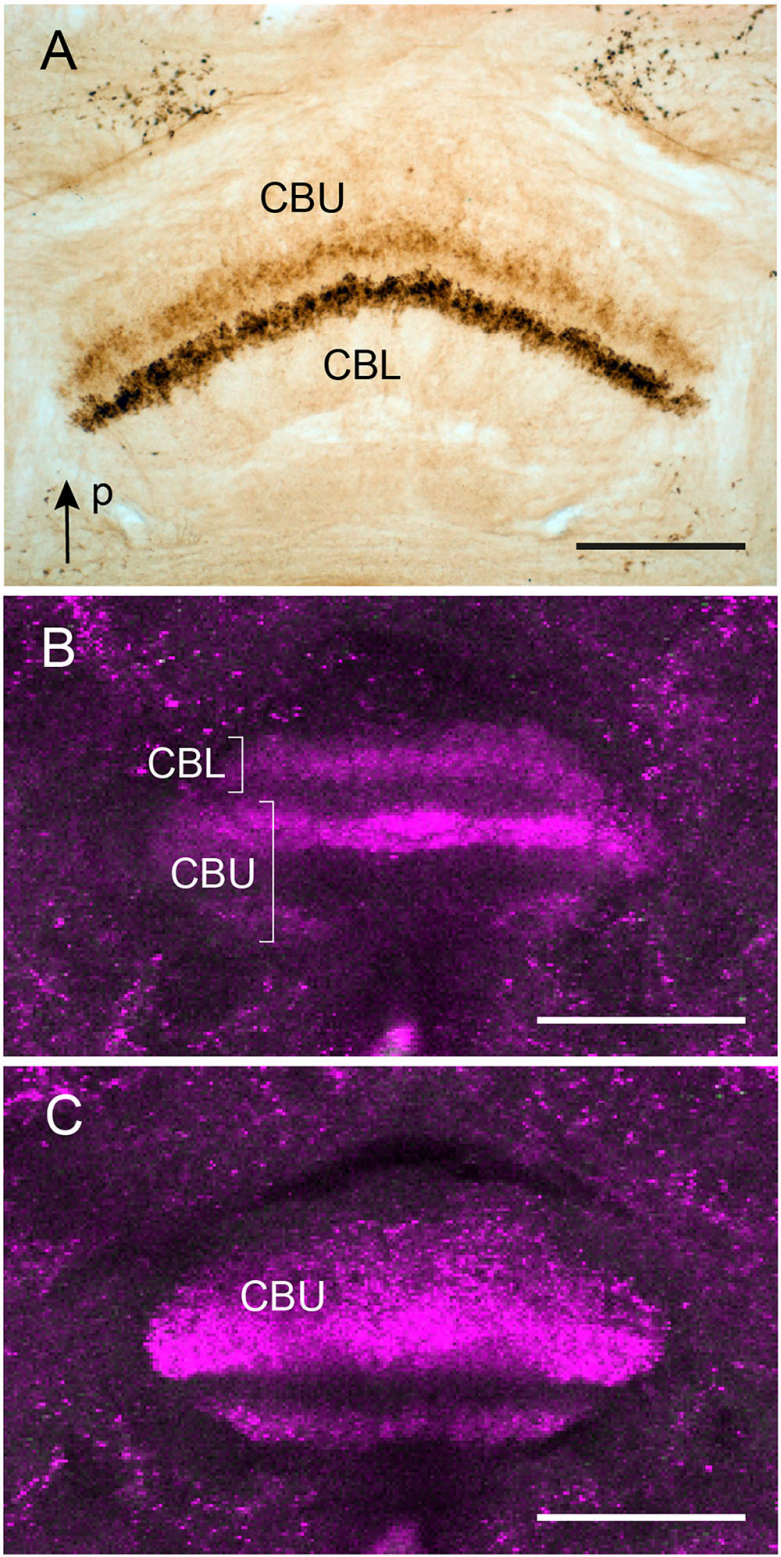
LomTK‐II immunostaining in the central body of the sphinx moth *Manduca sexta* (A) and the noctuid moth *Agrotis ipsilon* (B and C). (A) Horizontal peroxidase‐antiperoxidase labeled vibratome section. Two layers in the upper division of the central body (CBU) of *M. sexta* show LomTK‐II immunostaining, whereas the lower division of the central body (CBL) is free of staining. (B and C) Single frontal optical sections at an anterior (B) and posterior (C) level through the central body of *A. ipsilon* showing LomTK‐II immunofluorescence (magenta) in one layer of the CBL and two layers of the CBU. Scale bars = 100 µm. P, posterior.

Two lepidopteran species were studied, the tobacco hawk moth *M. sexta* (Sphingidae) and the noctuid *A. ipsilon* (Figure 12). In both species, particular layers of the central body show LomTK immunostaining, but in both species, the cellular origin of immunostaining in the central body could not be uncovered as no fibers entering or leaving the CBU were labeled. In *M. sexta*, two narrow layers of the CBU show immunostaining (Figure 12A). Beaded terminals in one layer in the CBU, immediately adjacent to the CBL, are intensely labeled, whereas a second layer, extending more posteriorly, is more faintly stained. The staining pattern confirms an earlier study showing LomTK‐I immunoreactivity in the central body of *M. sexta* (Skaer et al. 2002). In *A. ipsilon*, a median layer of the CBL and two layers in the CBU show immunolabeling (Figure 12B,C). In both species, the PB and the noduli are free of immunolabeling. Except for some large cell bodies of neurosecretory cells, no cell bodies are immunolabeled in the pars intercerebralis. The data in *A. ipsilon* are consistent with a study in the tobacco budworm *Heliothis virescens* (Noctuidae), where a basal layer of the CBU and a middle layer of the CBL showed LomTK‐II immunostaining (Zhao et al. 2017). Like in the two species studied here, immunostaining could not be traced to their cell bodies of origin.

### 3.11 Immunostaining in the CX of Flies (Diptera)

**FIGURE 13:**
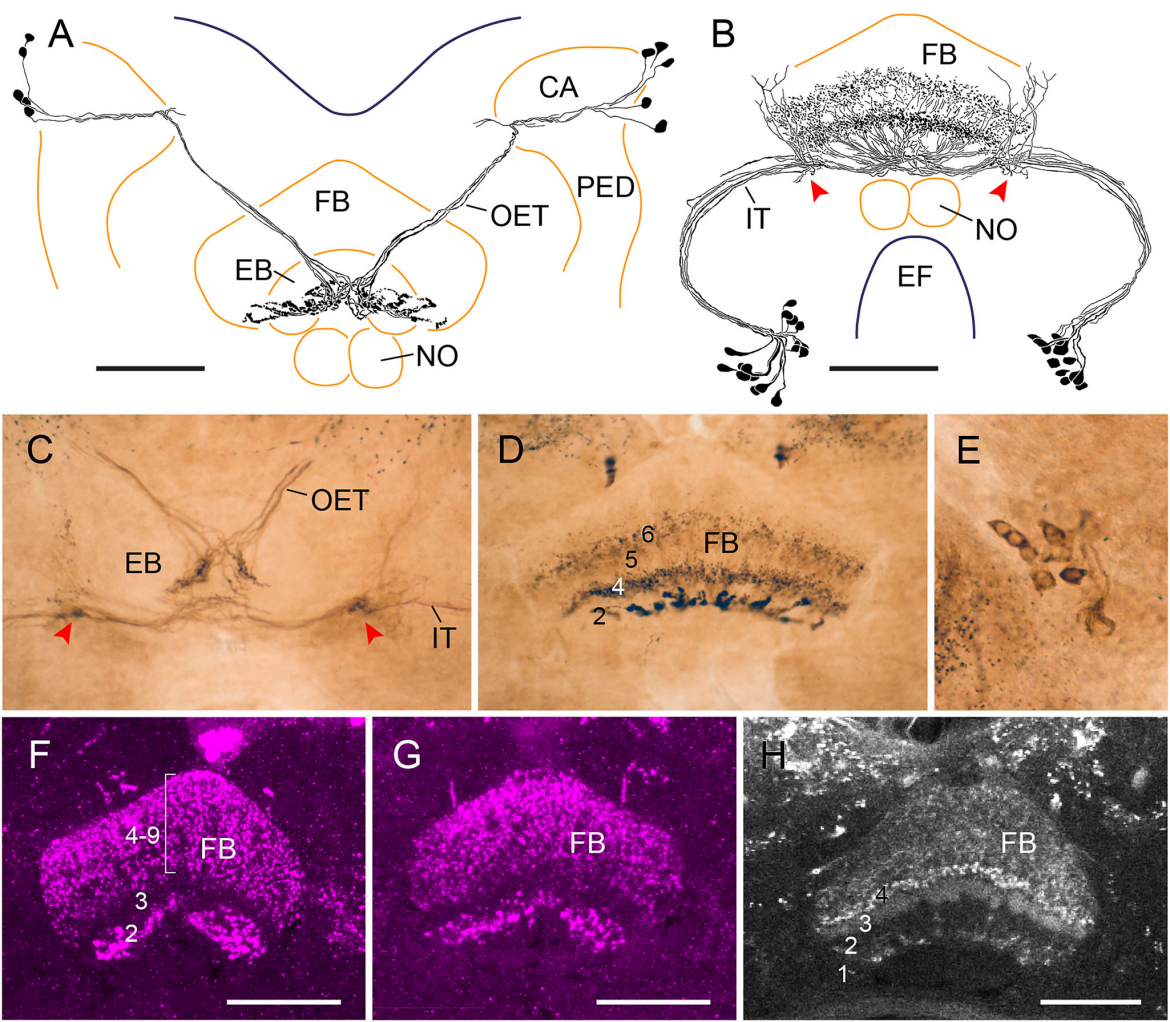
LomTK‐II immunostaining in the central complex of the blowfly *Calliphora vicina* (A–E), the fruit fly *Drosophila melanogaster* (F and G), and the crane fly *Tipula paludosa* (H). (A and B) Frontal reconstructions of two types of LomTK‐II‐immunolabeled tangential neuron innervating the fan‐shaped body (FB) of the blowfly *C. vicina*. (A) Five bilateral pairs of tangential LPP1 neurons with cell bodies near the calyces and proximal side branches in the superior protocerebrum send axonal fibers via the oblique ellipsoid tract (OET) and ventral groove of the ellipsoid body (EB) to varicose terminals in ventral layer 2 of the FB. (B) Twelve to sixteen tangential TC1 neurons with cell bodies near the vest sending principal neurites via the isthmus tract (IT) to beaded terminals in layers 4 and 6 of the FB. Before entering the FB, the neurons give off side branches toward superior brain areas (red arrowheads). (C) Image from two superimposed frontal sections showing the trajectories of LomTK‐II‐labeled fibers in the central body of the blowfly. Fibers from LPP1 neurons enter the FB via the OET and fibers from TC1 neurons, via the IT. Red arrowheads indicate sites of side branches sent off to superior brain areas (see panel B). (D) Frontal section through the FB of the blowfly. Three layers of the FB are immunolabeled, a ventral layer 2 with prominent varicose terminals from LPP1 neurons and intermediate layers 4 and 6 with terminals from TC1 neurons. (E) Frontal section showing cluster of TC1 cell bodies. (F and G) Stacks of frontal optical sections through the FB of the fruit fly *D. melanogaster* immunolabeled with the antiserum against LomTK‐I (F) and LomTK‐II (G). With both antisera, varicose terminals are labeled in ventral layer 2 of the FB, whereas beaded terminals extend across dorsal layers 4–9 of the FB. (H) Single optical section showing Lom‐TK‐I immunofluorescence in the FB of the crane fly *T. paludosa*. Four layers, labeled 1–4 and a broad superior band can be distinguished in the FB based on differences in immunolabeling. Fine terminals in layer 3 originate from LPP1‐type tangential neurons. Scale bars = 100 µm (A and B), 50 µm (C and D), 30 µm (E–G). CA, calyx; EB, ellipsoid body; EF, esophageal foramen; NO, noduli; PED, pedunculus.

Three dipteran species were studied, the blowfly *C. vicina*, the fruit fly *D. melanogaster*, and the European crane fly *T. paludosa*. In all three species, certain layers of the FB (equivalent to CBU) are immunolabeled, whereas the EB (equivalent to CBL), the noduli, and the PB are free of immunostaining (Figure 13). Immunostaining in *C. vicina* is consistent with earlier reports on immunolabeling with antisera against LomTK‐I and callitachykinin‐I in the CX of the blowfly *C. vomitoria* (Lundquist et al. 1994; Nässel et al. 1995). In both species, two types of tangential neuron of the CX, termed LPP1 (cell bodies in the posterior lateral protocerebrum) and TC1 (cell bodies in the anterior tritocerebrum) by Lundquist et al. (1994), exhibit immunostaining (Figure 13A,B). LPP1 neurons have cell bodies near the calyces of the mushroom body and dendritic ramifications in the superior protocerebrum. The neurons send axonal projections via the oblique ellipsoid tract and ventral groove of the EB to varicose terminals in ventral layer 2 of the FB (Figure 13A). Morphologically, these neurons correspond to FB2E–FB2J and/or FB2M neurons in *D. melanogaster* (Hulse et al. 2021) and TU_SLP_ neurons in the locust (von Hadeln et al. 2020). TC1 neurons have cell bodies near the vest (Figure 13B,E). Cell body fibers project in a wide arc via the isthmus tract to the FB. Before entering the FB, they give off side branches to the crepine and superior protocerebrum (Figure 13B,C). The neurons invade intermediate layers 4 and 6 of the FB (Figure 13B,D). Morphologically they correspond to TU_VES_4 neurons in the locust (von Hadeln et al. 2020) and FB4A and FB4D neurons targeting layer 4 of the FB and FB6N, FB6O, and FB6P neurons targeting layer 6 of the FB in *D. melanogaster* (Hulse et al. 2021).

Immunostaining for LomTK‐I and LomTK‐II yielded identical staining patterns in the FB of *D. melanogaster*. Like in *C. vicina*, layer 2 shows immunolabeled terminals as well as more dorsal layers 4–6, but in contrast to the blowfly, the most dorsal layers 7–9 are also immunolabeled (Figure 13F,G). These labeling patterns are consistent with earlier accounts using an antiserum against LemTRP‐1 from the cockroach *R. maderae* (Winther et al. 2003; Kahsai et al. 2010; Kahsai and Winther 2011). Immunostaining in layer 2 of the FB originates, like in the blowfly, from fibers passing through the central canal/ventral groove of the EB and, although they could not be traced to their cell bodies of origin, likely originate from LPP1 neurons as proposed by Nässel (2002). TC1 cell clusters are, likewise, immunolabeled in the *Drosophila* brain and might underly staining in the superior layers 4–6 of the FB (Winther et al. 2003). The presence of about 30–40 immunolabeled somata in the pars intercerebralis suggests that in addition certain pontine neurons (hΔ neurons) innervating the superior layers 7–9 exhibit LomTK immunostaining.

Immunolabeling in the crane fly *T. paludosa* is, as in the two other dipterans, confined to the FB, specifically layers 1, 3, 4, and all subsequent superior layers (Figure 13H). Fine terminals in layer 3 originate from prominent fascicles of neurons entering the FB via the oblique ellipsoid tract and ventral groove of the EB suggestive of LPP1‐type tangential neurons. The origin of labeling in all other layers could not be determined. Numerous small cell bodies are scattered in the pars intercerebralis, suggesting that certain types of columnar or pontine neurons may underly parts of the immunostaining in the central body.

## 4 Discussion

### 4.1 Evolution of LomTK Immunostaining in the CX

**FIGURE 14:**
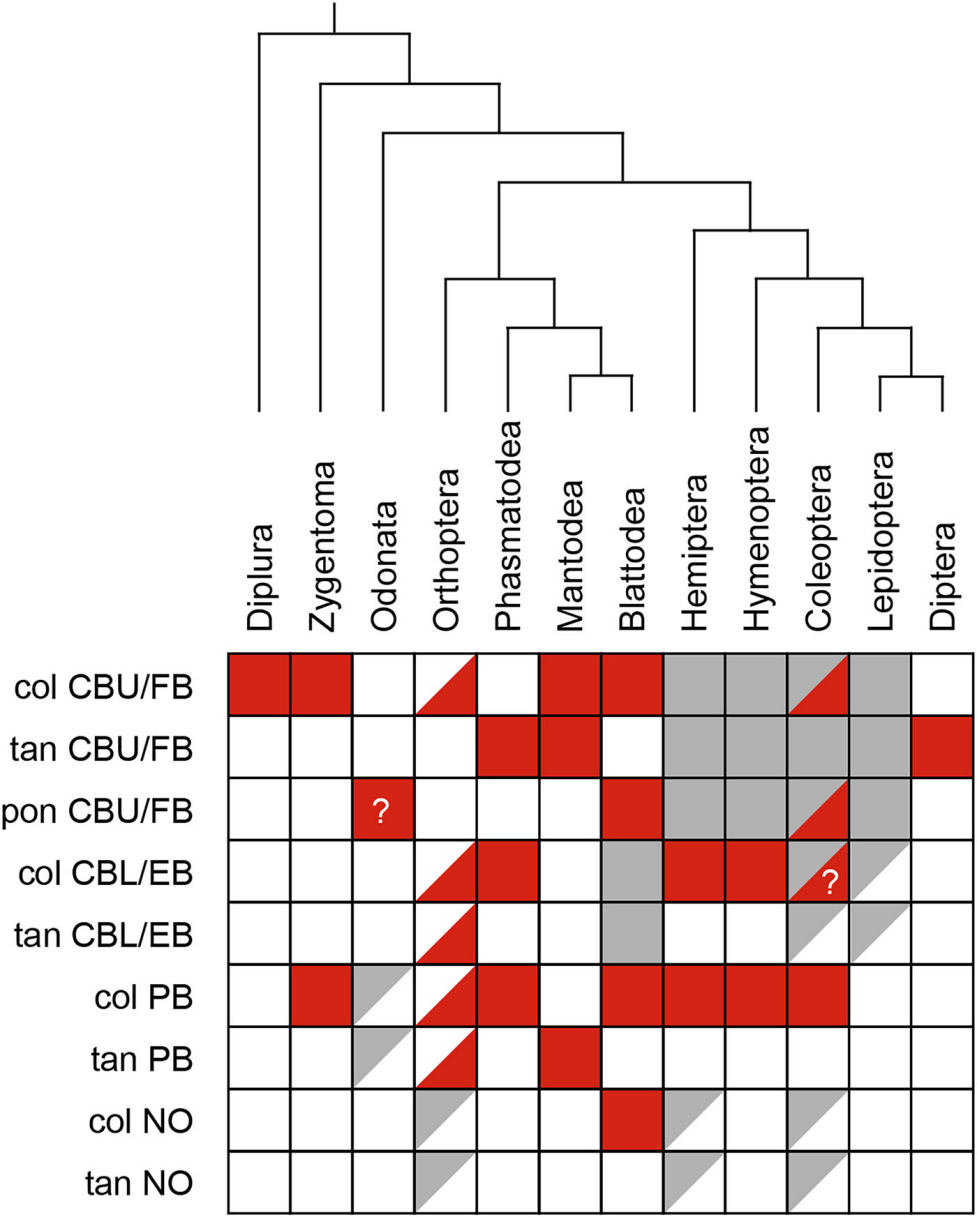
Occurrence of LomTK‐related peptides in the central complex (CX) of 12 hexapod orders, based on data in Table 2. Simplified evolutionary tree based on Misof et al. (2014). A full red square indicates the presence of LomTK peptides, a square half‐filled with red indicates presence in some, but not all, studied species of the order. A square filled in grey indicates the presence of LomTK peptides in the respective subunit of the CX without identification of the labeled cell type; a square half‐filled in grey indicates the presence of LomTKs in the respective subunit in some studied species, without identification of the labeled cell type. Question marks correspond to data in Table 2. CBL/EB, lower division of the central body/ellipsoid body; CBU/FB, upper division of the central body/fan‐shaped body; col, columnar neurons; NO, noduli; PB, protocerebral bridge; pon, pontine neurons; tan, tangential neurons.

Analysis of LomTK immunostaining in the CX of 25 hexapod species, ranging from two‐pronged bristletails to flies, reveals an unexpected diversity of immunolabeled cell types (Table 2). In some cases, even closely related clades differed in labeled cell types. Nevertheless, based on immunostaining in the two basal orders (Diplura, Zygentoma), immunolabeling of columnar neurons innervating the CBU/FB likely characterizes the ancestral state of tachykinin‐expressing neurons in the hexapod CX (Figure 14). Beginning with Zygentoma, representatives of all orders except Lepidoptera and Diptera also show tachykinin expression in the PB, usually in columnar neurons (Figure 14). Additional labeling of columnar neurons of the CBL/EB may be an apomorphy of Neoptera with secondary loss in Mantodea and the crown clades Lepidoptera and Diptera. Diptera (and possibly Lepidoptera) show the most striking transformations. Here, tachykinins are absent from columnar neurons and are instead expressed in tangential neurons of the FB (Table 2, Figure 14). In general, expression patterns are relatively clade‐specific, as illustrated in Odonata, Hymenoptera, Lepidoptera, and Diptera. However, substantial divergence was observed in some cases notably within Dictyoptera. In the two species studied, a cockroach (Blattodea) and a praying mantis (Mantodea), completely different types of columnar neurons were immunolabeled, including the sole incidence of columnar neurons innervating the noduli in the Madeira cockroach (Figure 14, Table 2). A particularly dramatic reduction has occurred in crickets, where tachykinins appear to be completely absent throughout the animal (Mochizuki et al. 2022). Whether the lack of tachykinins applies to all Gryllidae remains to be determined. Sequencing data from the Mormon cricket, *Anabrus simplex* (Tettigoniidae), suggest that tachykinin‐related peptides do exist in this branch of the Ensifera (NCBI Reference Sequence: XP_066998478.2).

**TABLE 2:** LomTK immunolabeling in the CX of the studied hexapods and possible homologous cell types in the locust *Schistocerca gregaria* and the fly *Drosophila melanogaster*.

Hexapod species	PB	CBU/FB	CBL/EB	NO	Locust type^a^	Fly type^b^
*Campodea augens*	−	++	−	−	CU	FC, FR
*Thermobia domestica*	++	++	−	−	CPU type^c^	PFG, PFR
*Platycnemis pennipes*	+	++	−	−	POUv^d^?	vΔ?
*Coenagrion puella*	−	++	−	−	POUv^d^?	vΔ?
*Libellula quadrimaculata*	−	++	−	−	POUv^d^?	vΔ?
*Schistocerca gregaria*	++	++	++	+	CU2x, CU2y CL1b TL2 TB6	FC types PEG ER2 −
*Acheta domesticus*	−	−	−	−		
*Medauroidea extradentata*	++	++	++	−	CL1c TU_VES_4	EPG, PEG FB4, FB6 type
*Rhyparobia maderae*	++	++	+	++	CP2 CPU5	PEG? PFN
*Hierodula membranacea*	+	++	−	−	CU2 TB type TU type	FC type Δ7 FB type
*Notonecta glauca*	++	+	++	−	CL1	EPG/PEG
*Graphosoma italicum*	++	+	++	+	CL1	EPG/PEG
*Apis mellifera*	+	++	++	−	CL1	PEG
*Vespula germanica*	+	++	++	−	CL1	PEG
*Vespula vulgaris*	+	++	++	−	CL1	PEG
*Tribolium castaneum*	+	++	+	−	CPU type^c^	PFG, PFR
*Zophobas morio*	+	++	+	−	CPU type^c^	PFG, PFR
*Acilius sulcatus*	+	++	++	+	CL type^e^ POUv^d^?	? vΔ?
*Gyrinus substriatus*	+	++	++	+	CL type^e^ POUv^d^?	? vΔ?
*Ilybius fuliginosus*	++	++	++	−	CL type^e^ POUv^d^?	? vΔ?
*Manduca sexta*	−	++	−	−	nd	nd
*Agrotis ipsilon*	−	++	+	−	nd	nd
*Calliphora vicina*	−	++	−	−	TU_SLP_ TU_VES_4	FB2 type FB4, FB6 type
*Drosophila melanogaster*	−	++	−	−	TU_SLP_ TU_VES_4 POUh	FB2 type FB4, FB6 type hΔ
*Tipula paludosa*	−	++	−	−	TU_SLP_	FB2 type

*Note:* ++, strong staining; +, weak/sparse staining; −, no staining.

Abbreviations: CBL/EB, lower division of the central body/ellipsoid body; CBU/FB, upper division of the central body/fan‐shaped body; nd, not determined; NO, noduli; PB, protocerebral bridge.

^a^ Based on Heinze and Homberg (2008), von Hadeln et al. (2020), Hensgen et al. (2021).

^b^ Based on Hulse et al. (2021).

^c^ CPU types with projections to the gall (PFG) or crepine (PFR) have not been described in the locust.

^d^ POUv neurons encountered only in the cockroach (Jahn et al. 2023).

^e^ CL types with bilateral projections have not been encountered in the locust.

### 4.2 Sequence Similarities and Staining Quality

The staining quality obtained with the two LomTK antisera varied depending on the species studied. Labeling was particularly poor in the odonates and lepidopterans and did not allow us to clearly identify the types of immunoreactive neurons. In general, staining quality may depend on the similarity of the species’ tachykinins with LomTK‐I and ‐II, on the concentration of processed tachykinins in all parts (cell bodies, dendrites, and axonal terminals) of the labeled neurons, but also on the presence of epitopes in the brain that affect background staining and might mask specific labeling. Judged from tachykinin sequence data (Table 1), no obvious species‐specific differences could be identified that might explain the differences in staining sensitivity. In addition to clearly identifiable cell types, immunostaining was often present at lower intensity in additional cell types that could not be identified clearly. Examples are tangential neurons of the CBU/FB in the stick insect (Figure 6D), labeling of the CBU/FB in hemipterans (Figure 8), and somata of neurons in the pars intercerebralis in the flightless hexapods, odonates, and flies that might target the CX. The underestimation of the extent of tachykinin expression by immunolabeling is particularly illustrated by a recent study in the fly *D. melanogaster* (Wolff et al. 2025). Through in situ hybridization of tachykinin gene expression in brains that expressed green fluorescent protein from a cell‐type specific split GAL4 line, the authors demonstrated the presence of tachykinins in pontine hΔD and hΔE neurons and in tangential FB2E, FB2I_a, FB2I_b, FB4O, and FB4R neurons. This list is likely incomplete, because cell‐type specific split GAL4 lines were not available for each cell type of the CX (e.g., FB6N and FB6O). Based on immunostaining using different antisera against tachykinins (Winther et al. 2003; Kahsai et al. 2010; Kahsai and Winther 2011; this study), layers 2 and 4–9 of the FB showed immunostaining as well as cell bodies in the pars intercerebralis, suggesting that tangential LPP1 neurons (probably corresponding to the FB2 subtypes), TC1 neurons (likely FB4 and FB6 subtypes), as well as neurons with cell bodies in the pars intercerebralis (likely hΔD and hΔE pontine neurons) express tachykinins. Taken together, these data suggest that the complete set of neuronal cell types that express tachykinins may also be larger in other species studied than the identified cell types reported here.

### 4.3 Diversity of Immunolabeled Columnar Neurons

Columnar neurons were LomTK‐immunoreactive in representatives of most hexapod orders, but the types of labeled neurons differed considerably and included at least five distinct cell types with ramifications in the CBU/FB or CBL/EB (Table 2). Although these differences in labeled cell types do not follow any simple evolutionary scenario, the LomTK‐labeled neurons in the two‐pronged bristletail and firebrat suggest that columnar neurons of the CBU/FB may represent the ancestral state, whereas LomTK‐labeled neurons of the CBL/EB might have occurred *de novo* in neopteran species. As shown in the fly *D. melanogaster*, the beetle *T. castaneum*, and the locust *S. gregaria*, columnar neurons of the CX originate developmentally from four bilateral pairs of type‐II neuroblasts in the pars intercerebralis (Boyan and Reichert 2011; Walsh and Doe 2017; Boyan et al. 2017; Farnworth et al. 2020). Each of these type‐II neural stem cells gives rise to lineages consisting of several hundred cells. The different types of neuron are born in a highly specific sequence. The morphological and neurochemical fate of the different sets of neurons is determined by temporal gradients of transcription factors and RNA binding proteins (Ren et al. 2017; Sullivan et al. 2019; Garcia‐Perez et al. 2021). Small changes in the expression levels of these factors can alter the number and morphology of their progeny as well as their neurotransmitter phenotype (Ren et al. 2017; Sullivan et al. 2019; Hamid et al. 2024). It is thus likely that various changes in the temporal expression of these regulatory genes have led to evolutionary changes in tachykinin expression in the different types of columnar and pontine neurons, serving the behavioral needs of the respective species.

Several aspects of the LomTK‐immunolabeled columnar neurons reveal both commonalities and species‐specific features in the internal organization of CX across hexapods. The universal presence of sets of 18 CL1/EPG/PEG‐type neurons in the locust, stick insect, heteropterans, hymenopterans (Sayre et al. 2021, this study), and a dragonfly (Homberg et al. 2023) suggests that, across insects, the PB consists of 18 columns as reported first in the fly *Drosophila* (Wolff et al. 2015) instead of 16 columns as was widely assumed before (e.g., Pfeiffer and Homberg 2014). The organization of the PB into 18 columns is further supported by the presence of 18 CP2 neurons in the cockroach (Figure 7). Based on their sparse ramifications in the CBL, these neurons may represent modified CL1 neurons connecting the PB, CBL, and gall. However, after passing through the posterior chiasma, their main fibers, unlike CL1 neurites, do not continue through the CBU toward the CBL but instead run along the dorso‐frontal surface of the CBU toward the anterior face of the CBL (Figure 7C).

A highly modified and reduced system of LomTK‐labeled CL1 neurons was found in the diving beetles. In *I. fuliginosus*, only three bilateral pairs of neurons are present. Their extensive ramifications in the PB, patchy innervation of the CBL, and bilateral projections to large areas, possibly corresponding to the gall in other species, are features not observed in other insects. Kollmann et al. (2016) showed that their antennal lobes show, likewise, several distinctive characteristics such as large numbers of miniature glomeruli, which together may represent adaptations to predatory aquatic lifestyle.

In many species the labeled columnar neurons, regardless of whether they innervate the CBL/EB or CBU/FB, project to small, in some cases highly focused, areas outside the CX. As shown in the fly *D. melanogaster* (Hulse et al. 2021), the locust *S. gregaria* (Heinze and Homberg 2008; Hensgen, Göthe, et al. 2021), and the cockroach *R. maderae* (Jahn et al. 2023), these areas lie in close proximity within the lateral complex and along the boundary between the crepine and lateral complex. They are termed gall, rubus, and round body and include parts of the crepine itself. In the two‐pronged bristletail and firebrat, three of these areas exhibit LomTK immunostaining (Figures 1A,D and 2B,D). The gall is innervated by CL1/EPG/PEG neurons, as well as by a specific type of CPU neuron termed PFG in the fly, which has not been encountered in the locust (Table 2). The round body in flies receives terminals from PFR neurons with arborizations in the PB and FB, but corresponding CPU‐type neurons have not yet been identified in other insect species. The rubus is targeted by a specific type of CU neuron termed FR in flies, and small parts of the crepine are innervated by CU neurons termed FC in flies (Table 2). In contrast to CX columnar outputs with large axonal ramifications (CPU1‐3 neurons in locusts, PFL neurons in flies), the gall, rubus, and round body appear to be largely involved in feedback loops within the CX network, as shown in flies (Hulse et al. 2021) and proposed in locusts (Hensgen, Göthe, et al. 2021). Their precise roles have not yet been elucidated.

### 4.4 Sparsely Immunolabeled Tangential Neurons

Only a few types of tangential neurons were LomTK‐immunolabeled in species from three orders (Table 2). They include tangential neurons of the PB in the locust and praying mantis and tangential neurons of the CBL and CBU in the locust (Table 2). In the fly *D. melanogaster* TU_VES_4 neurons correspond to FB tangential neurons derived from the BAmv1 lineage, such as FB4 and FB6 subtypes, and TU_SLP_ neurons to FB tangential neurons of the CP2 lineage, such as FB2 subtypes (Kandimalla et al. 2023). In contrast to the more basal hexapod orders, tangential neurons of the FB are the most strongly tachykinin‐immunolabeled neurons in flies and, based on the narrow layers immunolabeled in the central body, may also underly immunostaining in the lepidopteran species (Figure 12). The apparent lack of tachykinin expression in columnar neurons and the *de novo* expression in FB2E and FB2I tangential neurons in flies can thus be regarded as an apomorphy, perhaps shared by Diptera and Lepidoptera.

### 4.5 Functional Aspects

As shown in various insect species, the CX plays a key role in goal‐directed spatial orientation (Pfeiffer and Homberg 2014; Heinze 2023). Its columnar organization represents an internal compass architecture that receives input from celestial cues, wind direction, self‐generated proprioceptive feedback, and possibly efference copy signals. A large repertoire of tangential neurons of the CBU/FB modulates the overall activity of the CX based on internal needs and behavioral states. While the amines serotonin and dopamine, signaling substances largely present in CBU/FB tangential neurons (Timm et al. 2021; Homberg, Kirchner, et al. 2023), might mediate some of these needs and motivations, tachykinins, largely present in columnar neurons, might rather contribute to the fine tuning of compass properties and tuning aspects across insects. Specific functional roles of tachykinins in CX neurons have so far only been addressed in the fruit fly *D. melanogaster*, owing to the unique possibilities for genetic manipulation of defined neuron sets in this model organism. Kahsai et al. (2010) showed that RNAi‐mediated knockdown of tachykinin expression in neurons innervating the upper layers of the FB resulted in increased center‐zone avoidance in flies spontaneously walking in an arena. A role in sugar sensing and feeding preference was demonstrated for tachykinins released from vΔA_a pontine neurons in the dorsal FB (Musso et al. 2021). The neurons show oscillatory calcium activity when hemolymph glucose levels are high. Release of tachykinin inhibits postingestive fructose sensing, leading to a suppression of feeding. In both studies, tachykinins thus contribute to a modulation of orientation behaviors, a characteristic of FB/CBU function.

Although the general neuroarchitecture of the CX is highly conserved across insects (Pfeiffer and Homberg 2014; Heinze 2024), the present study shows that considerable differences exist in the distribution of certain neurotransmitters such as tachykinins. These findings suggest that functional studies in one species may not be easily generalized to all insect or hexapod species.

## Author Contributions

Study concept and design: Uwe Homberg. Acquisition of data: Jing Xu, Tim Keyser, Fedor Suzdalenkov, Fabian Wimmer, Martina Fromandi, Stefano Bianco, and Michelle Tez. Data analysis and interpretation: Jing Xu, Tim Keyser, Fedor Suzdalenkov, Fabian Wimmer, Stefan Dippel, Stefano Bianco, Michelle Tez, Martina Fromandi, and Uwe Homberg. Drafting the manuscript, review and editing: Uwe Homberg, Stefan Dippel.

## Ethics Statement

All animal procedures were performed according to the guidelines of the European Union (Directive 2010/63/EU) and the German Animal Welfare Act. Permission to collect insects near the Department of Biology was obtained from Untere Naturschutzbehörde Marburg.

## Conflicts of Interest

Dr. Uwe Homberg is an Editorial Board member of this submitted JCN Journal and the corresponding author of this article. To minimize bias, they were excluded from all editorial decision‐making related to the acceptance of this article for publication.

## Data Availability

All relevant data are available from the corresponding author.
